# Development of CRISPR/Cas9-mediated gene disruption systems in *Giardia lamblia*

**DOI:** 10.1371/journal.pone.0213594

**Published:** 2019-03-11

**Authors:** Zi-Qi Lin, Soo-Wah Gan, Szu-Yu Tung, Chun-Che Ho, Li-Hsin Su, Chin-Hung Sun

**Affiliations:** Department of Parasitology, College of Medicine, National Taiwan University, Taipei, Taiwan, ROC; Aga Khan University - Kenya, KENYA

## Abstract

*Giardia lamblia* becomes dormant by differentiation into a water-resistant cyst that can infect a new host. Synthesis of three cyst wall proteins (CWPs) is the fundamental feature of this differentiation. Myeloid leukemia factor (MLF) proteins are involved in cell differentiation, and tumorigenesis in mammals, but little is known about its role in protozoan parasites. We developed a CRISPR/Cas9 system to understand the role of MLF in *Giardia*. Due to the tetraploid genome in two nuclei of *Giardia*, it could be hard to disrupt a gene completely in *Giardia*. We only generated knockdown but not knockout mutants. We found that knockdown of the *mlf* gene resulted in a significant decrease of *cwp* gene expression and cyst formation, suggesting a positive role of MLF in encystation. We further used *mlf* as a model gene to improve the system. The addition of an inhibitor for NHEJ, Scr7, or combining all cassettes for gRNA and Cas9 expression into one plasmid resulted in improved gene disruption efficiencies and a significant decrease in *cwp* gene expression. Our results provide insights into a positive role of MLF in inducing *Giardia* differentiation and a useful tool for studies in *Giardia*.

## Introduction

*Giardia lamblia* is a common cause of waterborne diarrhea due to contamination of water with human or animal feces [1,2]. Although most cases have self-limiting diarrhea, a subset of patients may develop chronic giardiasis or irritable bowel syndrome after giardiasis [3]. Chronic giardiasis in children may lead to malnutrition and cognitive impairment [4–6]. *Giardia* can serve as a unique protozoan model for eukaryotic cell differentiation because its life cycle has been completed with the test tube [1,7]. Like many other organisms that adapt to inhospitable environments by persisting in a dormant state, *G*. *lamblia* undergoes differentiation from a pathogenic trophozoite form into a resistant infectious cyst form [1,8]. The cysts are protectively walled by cyst wall proteins (CWPs) and resistant to hypotonic lysis by fresh water and gastric acid [9–11]. Synthesis of at least two CWPs is coordinately induced during encystation, possibly by signaling molecules and transcription factors, including CDK2, MYB2, WRKY, PAX1, and E2F1 [12–16]. Histone modifications or epigenetic mechanisms may be involved in CWP expression and cyst formation [17,18].

Stable transfection systems that are involved in maintenance of episomal plasmids under drug selection, including neomycin and puromycin selection have been developed in *Giardia* for a stable and high expression of a target gene [19,20]. A system for knockout of a target gene in *Giardia* for studies of gene function is also needed. A Cre/loxP system has been developed for genetic manipulations [21,22], but it is tedious to have a complete knockout in all alleles, because of a tetraploid genome in two nuclei of *Giardia* [23]. Although a virus-based antisense ribozyme or a dsRNA vector for mRNA knockdown has been reported [24,25], they are not widely used. Morpholinos have been used to knockdown gene expression but they are very expensive [26]. Development of a CRISPR/Cas9 system is in urgent need for studies of *Giardia*. A CRISPRi system with catalytically inactive Cas9 has been demonstrated for stable transcriptional repression of flagellar and ventral disc genes in *Giardia* recently [27]. CRISPR/Cas9 systems have been developed in several protozoan parasites, including *Plasmodium*, *Toxoplasma*, *Trypanosoma*, *Leishmania*, and *Trichomonas* [28–32].

The type II CRISPR/Cas9 system is from *Streptococcus pyogenes* for defense against viral or plasmid DNA invasion [33]. Cas9 can form a complex with gRNA to target DNA by recognizing a protospacer adjacent motif (PAM), which has NGG sequence [34,35]. After cleavage of DNA by Cas9/gRNA complex, dsDNA break will form (34, 35). To repair DNA break, cells may use an “error-prone” non-homologous end joining (NHEJ) or an “error-free” homologous recombination (HR) pathway [36]. NHEJ generates 1 to 10 bp insertion or deletion and subsequent frameshift mutations at the cleavage site, thereby introducing gene mutation or knockout [36,37]. HR has lower frequency [37,38], but enables error free targeted gene knockin. Knocking in of a DNA cassette allows introducing insertion of a drug resistance gene when an HR template cassette with two homology arms are present [37]. HR-dependent target gene replacement by knocking in of a drug resistant gene can lead to target gene disruption in the CRISPR/Cas9 system [28,37]. Scr7 has been used in mammalian cells to increase knock-in efficiency via CRISPR/Cas9-coupled HR [39,40, 41]. Scr7 inhibits NHEJ by blocking ligase IV mediated joining of dsDNA break and is shown to increase efficiency of precise genome editing by CRISPR/Cas9 in mammalian cells [40].

The myeloid leukemia factor (*mlf*) genes have been identified only in mammals and *Drosophila* [42,43], but not in yeast or plants. Human MLF1 is important for normal hemopoietic differentiation and its dysregulation may cause leukemia [42,44,45]. Human MLFs play a role in maintaining protein stability or in interacting with a secreted protein, secretagogin, or Cop9 signalosome, a proteasome-regulatory system [43,46,47]. Little is known about the role of MLFs in mammals or in protozoan parasites. A MLF-like protein has been identified in the *Giardia* genome with a higher expression level during drug selection [48]. The *mlf* gene is upregulated during encystation [48], suggesting a demand for MLF protein increases during encystation. To date, no member of this gene family has been reported in protozoan parasites.

Since mammalian MLF proteins play critical roles in cell differentiation, we tried to understand the role of MLF in *Giardia* differentiation into cysts. We found that overexpression of MLF can increase the *cwp1-3* and *myb2* gene expression and cyst formation. We then developed a CRISPR/Cas9 system with two plasmids expressing Cas9 and gRNA separately. We found that knockdown of *mlf* gene by the CRISPR/Cas9 system resulted in a significant decrease of *cwp* gene expression and cyst formation. We used this first successful target gene, *mlf*, as a model to design an improved CRISPR/Cas9 system. We found that addition of Scr7, an inhibitor for NHEJ, resulted in a significantly improved gene disruption efficiency. In addition, the combination of all cassettes into one plasmid resulted in a further improved gene disruption efficiency. Our newly CRISPR/Cas9 system provides positive evidence of MLF in inducing *Giardia* differentiation into cysts and can be exploited to down regulate gene expression and study important functions of genes in *G*. *lamblia*.

## Material and methods

### *G*. *lamblia* culture

Trophozoites of *G*. *lamblia* WB, clone C6 (see ATCC 50803)(obtained from ATCC), were cultured in modified TYI-S33 medium [49]. Encystation was performed as previously described [11]. Briefly, trophozoites grown to late log phase in growth medium were harvested and encysted for 24 h in TYI-S-33 medium containing 12.5 mg/ml bovine bile at pH 7.8 at a beginning density of 5×10^5^ cells/ml.

### Cyst count

Cyst count was performed on the stationary phase cultures (~2×10^6^ cells/ml) during vegetative growth as previously described [48,50]. The cells were subcultured in growth medium with suitable selection drugs at an initial density of 1×10^6^ cells/ml. Cells seeded at this density became confluent within 24h. Confluent cultures were maintained for an additional 8h to ensure that the cultures were in stationary phase (at a density of ~2×10^6^ cells/ml). Cyst count was performed on these stationary phase cultures. Cyst count was also performed on 24h encysting cultures.

### Isolation and analysis of the *mlf* gene

Synthetic oligonucleotides used are shown in S1 Table. The *mlf* coding region with 324 bp of 5’- flanking region was cloned and the nucleotide sequence was determined. The *mlf* gene sequence in the database was correct. To isolate the cDNA of the *mlf* gene, we performed RT-PCR with *mlf*-specific primers using total RNA from *G*. *lamblia*. For RT-PCR, 5 μg of DNase-treated total RNA from vegetative and 24 h encysting cells was mixed with oligo (dT)12-18 and random hexamers and Superscript II RNase H- reverse transcriptase (Invitrogen). Synthesized cDNA was used as a template in subsequent PCR with primers mlfF and mlfR. Genomic and RT-PCR products were cloned into pGEM-T easy vector (Promega) and sequenced (Applied Biosystems, ABI). Comparison of genomic and cDNA sequences showed that the *mlf* gene contained no introns.

### Genomic DNA extraction, PCR and quantitative real-time PCR analysis

Synthetic oligonucleotides used are shown in S1 Table. Genomic DNA was isolated from trophozoites using standard procedures [51]. For PCR, 250 ng of genomic DNA was used as a template in subsequent PCR. PCR analysis of *mlf* (**XM_001706985.1**, open reading frame 16424), *cwp1* (**U09330**, open reading frame 5638), *cwp2* (**U28965**, open reading frame 5435), and *ran* (**U02589**, open reading frame 15869) genes was performed using primers mlfF (PCR1F) and mlfR (PCR1R), PCR2F and PCR2R, PCR3 and PCR3R, cwp1F and cwp1R, cwp2F and cwp2R, ranF and ranR, respectively. For quantitative real-time PCR, SYBR Green PCR master mixture was used (Kapa Biosystems). PCR was performed using an Applied Biosystems PRISMTM 7900 Sequence Detection System (Applied Biosystems). Specific primers were designed for detection of the *mlf*, *cwp1*, *cwp2*, and *ran* genes: mlfrealF and mlfrealR; cwp1realF and cwp1realR; cwp2realF and cwp2realR; ranrealF and ranrealR. Two independently generated stably transfected lines were made from each construct and each of these cell lines was assayed three separate times. The results are expressed as a relative expression level over control. Student’s *t*-tests were used to determine statistical significance of differences between samples.

### RNA extraction, RT-PCR and quantitative real-time PCR analysis

Synthetic oligonucleotides used are shown in S1 Table. Total RNA was extracted from *G*. *lamblia* cell line during vegetative growth or encystation stages using TRIzol reagent (Invitrogen). For RT-PCR, 5 μg of DNase-treated total RNA was mixed with oligo (dT)12-18 and random hexamers and Superscript II RNase H^-^ reverse transcriptase (Invitrogen). Synthesized cDNA was used as a template in subsequent PCR. Semi-quantitative RT-PCR analysis of *mlf* (**XM_001706985.1**, open reading frame 16424), *mlf-ha*, *cwp1* (**U09330**, open reading frame 5638), *cwp2* (**U28965**, open reading frame 5435), *cwp3* (**AY061927**, open reading frame 2421), *myb2* (**AY082882**, open reading frame 8722), *wrky* (**XM_001708755**, open reading frame 9237), *pax1* (**XM_001704983**, open reading frame 32686), *cdk2* (**XP_001709931.1**, open reading frame 16802), gRNA, *ran* (**U02589**, open reading frame 15869), and 18S ribosomal RNA (**M54878**, open reading frame r0019) gene expression was performed using primers mlfF and mlfR, mlfHAF and HAR, cwp1F and cwp1R, cwp2F and cwp2R, cwp3F and cwp3R, myb2F and myb2R, wrkyF and wrkyR, pax1F and pax1R, cdk2F and cdk2R, MLFgF and MLFgR, ranF and ranR, 18SrealF and 18SrealR, respectively. For quantitative real-time PCR, SYBR Green PCR master mixture was used (Kapa Biosystems). PCR was performed using an Applied Biosystems PRISMTM 7900 Sequence Detection System (Applied Biosystems). Specific primers were designed for detection of the *mlf*, *cwp1*, *cwp2*, *cwp3*, *myb2*, *wrky*, *pax1*, *cdk2*, *ran*, and 18S ribosomal RNA genes: mlfrealF and mlfrealR; cwp1realF and cwp1realR; cwp2realF and cwp2realR; cwp3realF and cwp3realR; myb2realF and myb2realR; wrkyrealF and wrkyrealR; pax1realF and pax1realR; cdk2realF and cdk2realR; ranrealF and ranrealR; 18SrealF and 18SrealR. Each primer pairs were determined for amplification efficiency ~95% based on the slope of the standard curve. Two independently generated stably transfected lines were made from each construct and each of these cell lines was assayed three separate times. The results are expressed as a relative expression level over control. Student’s *t*-tests were used to determine statistical significance of differences between samples.

### Plasmid construction

Synthetic oligonucleotides used are shown in S1 Table. All constructs were verified by DNA sequencing with a BigDye Terminator 3.1 DNA Sequencing kit and an Applied Biosystems 3100 DNA Analyszer (Applied Biosystems). Plasmid 5’Δ5N-Pac was a gift from Dr. Steven Singer and Dr. Theodore Nash [20]. Plasmids pRANneo has been described previously [19]. The 227-bp 5’-flanking region of *mlf* gene was amplified with oligonucleotides MLF 5HF and MLF 5NR, digested with *Hind*III/*Nco*I and cloned into *Hind*III/*Nco*I digested 5’Δ5N-Pac, resulting in MLF5. The 700-bp 3’-flanking region of *mlf* gene was amplified with oligonucleotides MLF 3XF and MLF 3KR, digested with *Xba*I/*Kpn*I and cloned into *Xba*I/*Kpn*I digested MLF5, resulting in MLF53. We used gene synthesis services from IDT to obtain the fragment MLF-guide. The NCBI Nucleotide Blast search was used to avoid the potential off-target effects of guide sequence. The MLF-guide was digested with *Kpn*I/*Eco*RI and cloned into *Kpn*I/*Eco*RI digested MLF53, resulting in pMLFko. Cas9 gene was amplified using primers Cas9NF and Cas9XR with the template pCas9_GFP (Addgene), digested with *Nco*I/*Xho*I and cloned into *Nco*I/*Xho*I digested 5’Δ5N-Pac, resulting in pgCas9dC. We used gene synthesis services from IDT to obtain the fragment Cas9CXK. The Cas9CXK was digested with *Xho*I/*Kpn*I and cloned into *Xho*I/*Kpn*I digested pgCas9dC, resulting in pgCas9.

To make construct pPMLF, the *mlf* gene and its 324 bp of 5’- flanking region were amplified with oligonucleotides MLFNF and MLFMR, digested with NheI/MluI, and cloned into NheI/MluI digested pPop2NHA [52]. To make pU6g, the MLF-guide was digested with *Kpn*I/*Eco*RI and cloned into *Kpn*I/*Eco*RI digested pBluescript SK, resulting in pgU6. To make pgCas9MLFko, Q5 Site Directed Mutagenesis Kit was used to perform a reaction with a pgCas9 template, and primers Cas9insF and Cas9insR to insert multiple restriction sites, resulting in pgCas9ins. The HR template (MLF5’, *pac*, and MLF3’) and gRNA was amplified with the pMLFko plasmid and primers, mlf53UNF and mlf53UMR, digested with *Not*I/*Mlu*I and cloned into *Not*I/*Mlu*I digested pgCas9ins, resulting in pgCas9MLFko. To make pgCas9MLFko gRNA1, Q5 Site Directed Mutagenesis Kit was used to perform a reaction with a pgCas9MLFko template, and primers U6R and MLFgFnew to change the gRNA, resulting in pgCas9MLFko gRNA1. To make pgCas9MLFko gRNA2, Q5 Site Directed Mutagenesis Kit was used to perform a reaction with a pgCas9MLFko template, and primers U6R and control gF to change the gRNA, resulting in pgCas9MLFko gRNA2.

### Transfection and Western blot analysis

Cells transfected with pPMLF plasmid containing the *pac* gene were selected and maintained with 54 μg/ml (100 μM) puromycin [20]. For CRISPR/Cas9 system, *Giardia* trophozoites were transfected with plasmid pMLFko and pgCas9, and then selected in 100 μM puromycin. The MLFko stable transfectants were established after selection. Stable transfectants were maintained at 100 μM puromycin and were further analyzed by Western blot, or DNA/RNA extraction. The replacement of the *mlf* gene with *pac* gene was confirmed by PCR and sequencing. The control is *G*. *lamblia* trophozoites transfected with double amounts of 5’Δ5N-Pac plasmid and selected with puromycin. For removal of puromycin experiments, puromycin was removed from the medium for each stable cell line for a month to obtain MLFko–pu and control–pu cell line. For establishing MLFkoSC stable transfectants, the same plasmids were used, except that the culture medium in the first replenishment contained 6 μM Scr7 and 100 μM puromycin. For establishing Cas9MLFko stable transfectants, *Giardia* trophozoites were transfected with plasmid pgCas9MLFko, and selected in 25 μM puromycin. The culture medium in the first replenishment contained 1.5 μM Scr7 and 25 μM puromycin. The Cas9MLFko stable transfectants were established after selection. Stable transfectants were maintained at 100 μM puromycin and were further analyzed by Western blot, or DNA/RNA extraction. The control cell line is *G*. *lamblia* trophozoites transfected with 5’Δ5N-Pac plasmid and selected with puromycin. Puromycin was then removed from the medium for each stable cell line to obtain Cas9MLFko–pu cell line. Subsequent analysis was performed after removal of the drug for a month. The control cell line is wild type nontransfected WB trophozoites. The single cell population was obtained by dilution of the Cas9MLFko cell line. The cell density was determined first and then diluted to 1 cell/culture tube. The single cell populations were established within 2 weeks.

Western blots were probed with anti-HA monoclonal antibody (1/5000 in blocking buffer; Sigma), anti-MLF (1/10000 in blocking buffer) (see below), anti-CWP1 (1/10000 in blocking buffer) [13], anti-MYB2 (1/5000 in blocking buffer) [16], anti-WRKY (1/5000 in blocking buffer) [14], anti-PAX1 (1/10000 in blocking buffer)[15], anti-CDK2 (1/10000 in blocking buffer)[12], anti-RAN (1/10000 in blocking buffer)[53], or preimmune serum (1/5000 in blocking buffer), and detected with peroxidase-conjugated goat anti-mouse IgG (1/5000; Pierce) or peroxidase-conjugated goat anti-rabbit IgG (1/5000; Pierce) and enhanced chemiluminescence (Millipore).

### Expression and purification of recombinant MLF protein

The genomic *mlf* gene was amplified using oligonucleotides mlfF and mlfR. The product was cloned into the expression vector pET101/D-TOPO (Invitrogen) in frame with the C-terminal His and V5 tag to generate plasmid pMLF. The pMLF plasmid was freshly transformed into *Escherichia coli* BL21 Star (DE3) (Invitrogen). An overnight pre-culture was used to start a 250-ml culture. *E*. *coli* cells were grown to an A600 of 0.5, and then induced with 1mM isopropyl-D-thiogalactopyranoside (IPTG) (Promega) for 4 h. Bacteria expressing pMLF were harvested by centrifugation and sonicated in 10 ml of buffer A (100 mM sodium phosphate, 10mM Tris-Cl, 6M Guanidine Hydrochloride, pH8.0) containing 10 mM imidazole and complete protease inhibitor cocktail (Roche). The samples were centrifuged and the supernatant was mixed with 1 ml of a 50% slurry of Ni-NTA superflow (Qiagen). The resin was washed with buffer B (100 mM sodium phosphate, 10mM Tris-Cl, 8M urea, pH8.0) and buffer C (100 mM sodium phosphate, 10mM Tris-Cl, 8M urea, pH6.3) and eluted with buffer E (100 mM sodium phosphate, 10mM Tris-Cl, 8M urea, pH4.5). Fractions containing MLF were pooled, dialyzed in 25 mM HEPES pH 7.9, 40 mM KCl, and 15% glycerol, and stored at -70°C. Protein purity and concentration were estimated by Coomassie Blue and silver staining compared with bovine serum albumin. MLF protein is purified to apparent homogeneity (>95%).

### Generation of anti-MLF antibody

Purified MLF protein was used to generate rabbit polyclonal antibodies through a commercial vendor (Angene, Taipei, Taiwan).

### Immunofluorescence assay

Cells cultured in growth medium or encystation medium for 24 h were harvested, washed in phosphate-buffered saline (PBS), and attached to glass coverslips (2 × 10^6^ cells/coverslip) and then fixed and stained [54]. Cells were reacted with anti-MLF, or anti-HA monoclonal antibody (1/300 in blocking buffer; Sigma). Anti-rabbit ALEXA 568 or anti-mouse ALEXA 488 (1/500 in blocking buffer, Life Technologies) was used as the detector. ProLong antifade kit with 4’,6-diamidino-2-phenylindole (Life Technologies) was used for mounting. MLF or Cas9-HA protein was visualized using a Leica TCS SP5 spectral confocal system. Images were analyzed by Imaris software (Bitplane).

## Results

### MLF induced the expression of *cwp1-3* and *myb2* genes

Previous studies have shown that a gene encoding a MLF-like protein is up-regulated significantly during encystation and its gene promoter has the binding sites of the MYB2 transcription factor and is up-regulated by MYB2 [13,48], suggesting that MLF could be important for *Giardia* encystation. The deduced *Giardia* MLF protein contains 252 amino acids with a predicted molecular mass of ~29.71 kDa and a pI of 6.81. It has one MLF1IP domain (residues 9 to 170) as predicted by Pfam (or residues or 59 to 170 by NCBI Conserved Domain Database) (S1 Fig). The MLFIP domain can also be found in human MLF1 (residues 26 to 202), MLF2 (residues 10 to 199), and *Drosophila* MLF (residues 36 to 199). The *Drosophila* MLF1IP domain contains a 14-3-3 binding motif, which may involve in interaction with the transcription factor Dref [44], but the *Giardia* MLFIP domain has no 14-3-3 interaction motif, RSXSXPRXSXSX [44]. Sequence alignment shows that *Giardia* MLF is moderately similar to the human MLF1 and MLF2 and *Drosophila* MLF (S1 Fig). The full-length of *Giardia* MLF has 12.69% identity and 24.63% similarity to that of human MLF1 (calculated from S1 Fig).

**Fig 1:**
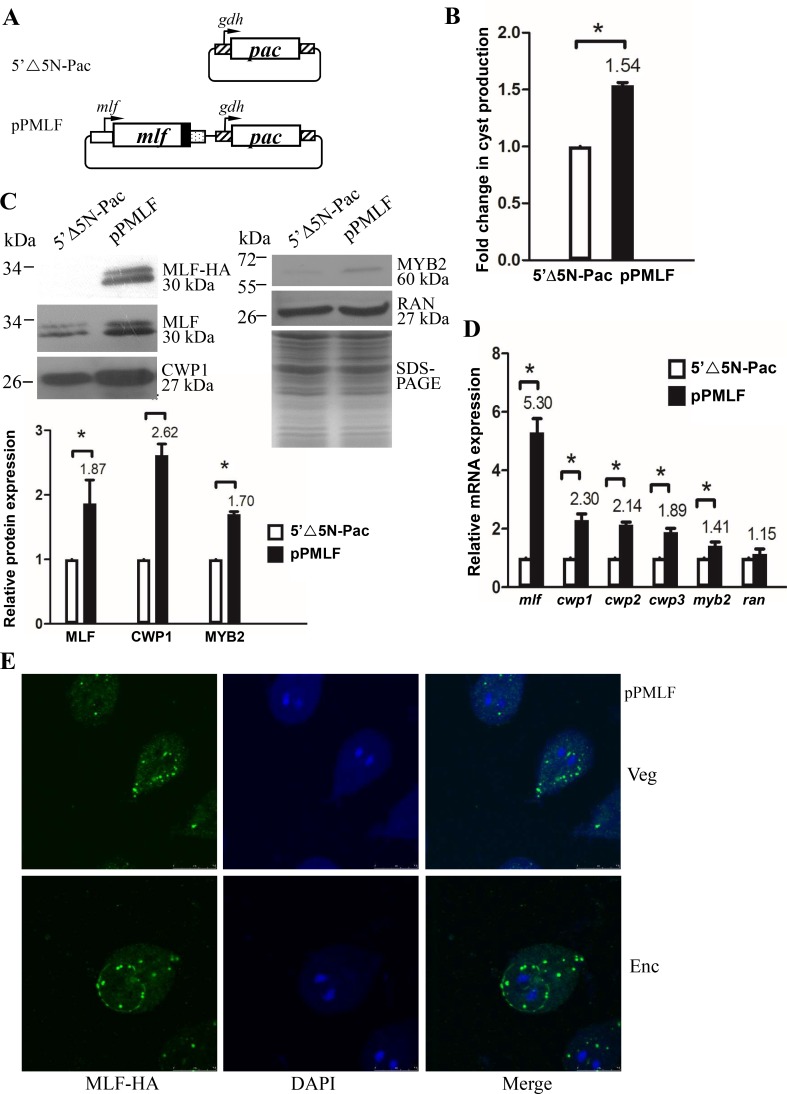
Induction of *cwp1-3* and *myb2* gene expression in the MLF overexpressing cell line. (A) Diagrams of the 5’Δ5N-Pac and pPMLF plasmid. The *pac* gene (open box) is under the control of the 5’- and 3’-flanking regions of the *gdh* gene (striated box). In construct pPMLF, the *mlf* gene is under the control of its own 5’-flanking region (open box) and the 3’-flanking region of the *ran* gene (dotted box). The filled black box indicates the coding sequence of the HA epitope tag. (B) MLF overexpression increased cyst formation. The 5’Δ5N-Pac and pPMLF stable transfectants were cultured in growth medium and then subjected to cyst count as described under “Material and Methods”. The sum of total cysts is expressed as relative expression level over control. Values are shown as means ± S. E. *, P <0.05. (C) Overexpression of MLF increased the levels of CWP1 and MYB2 proteins. The 5’Δ5N-Pac and pPMLF stable transfectants were cultured in growth medium and then subjected to SDS-PAGE and Western blot analysis. The blot was probed with anti-HA, anti-MLF, anti-CWP1, anti-MYB2, and anti-RAN antibodies, respectively. Equal amounts of protein loading were confirmed by SDS-PAGE and Coomassie Blue staining. A similar level of the RAN protein was detected. The intensity of bands from three Western blot assays was quantified using Image J. The ratio of each target protein over the loading control RAN is calculated. Fold change is calculated as the ratio of the difference between pPMLF cell line and the control cell line, to which a value of 1 was assigned. Results are expressed as mean ± SD. *P<0.05. (D) Quantitative real-time PCR analysis of gene expression in the MLF-overexpressing cell lines. The 5’Δ5N-Pac and pPMLF stable transfectants were cultured in growth medium and then subjected to quantitative real-time PCR analysis using primers specific for *mlf*, *cwp1*, *cwp2*, *cwp3*, *myb2*, *ran*, and 18S ribosomal RNA genes, respectively. Transcript levels were normalized to 18S ribosomal RNA levels. Fold changes in mRNA expression are shown as the ratio of transcript levels in the pPMLF cell line relative to the 5’Δ5N-Pac cell line. Results are expressed as the means ± S. E. of at least three separate experiments. *, P <0.05. Similar levels of the *ran* mRNAs were detected. (E) Localization of MLF. The pPMLF stable transfectants were cultured in growth (Veg, vegetative growth, upper panel) or encystation medium for 24 h (Enc, encystation, lower panel) and then subjected to immunofluorescence analysis using anti-HA antibody for detection. The left panel shows that the MLF-HA protein is localized to the MLF-vesicles (MVs) in the cytoplasm of vegetative and encysting trophozoites. MLF also localized to sucking disc edge in an encysting trophozoite. The middle panels show the DAPI staining of cell nuclei and differential interference contrast images. The right panel shows the merged images.

To determine the role of MLF protein, we prepared construct pPMLF, in which the *mlf* gene is controlled by its own promoter and contains an HA epitope tag at its C terminus (Fig 1A) and stably transfected it into *Giardia*. We first investigated the effect of MLF on cyst formation. In previous studies, we obtained consistent cyst number data from vegetative *G*. *lamblia* cultures during growth to stationary phase due to spontaneous differentiation [48]. In this study, we found that the cyst number in the MLF overexpressing cell line significantly increased relative to the control cell line (Fig 1B). MLF-HA protein was detected as a ~30kDa protein with anti-HA antibody, consistent with the predicted 30kDa molecular mass of MLF with the HA tag (1 kDa)(Fig 1C). Overexpression of MLF in the pPMLF cell line also can be confirmed by the anti-MLF antibody at a size of ~30 kDa (Fig 1C and S2 Fig). MLF overexpression resulted in a significant increase of the CWP1 and MYB2 protein levels (Fig 1C). Quantitative real-time PCR and RT-PCR analysis showed that the mRNA levels of the endogenous *mlf* plus vector expressed *mlf* in the MLF overexpressing cell line significantly increased relative to the vector control cell line (Fig 1D and S2 Fig). The levels of the *cwp1-3* and *myb2* mRNAs in the MLF overexpressing cell line also significantly increased relative to the vector control cell line (Fig 1D and S2 Fig). Our results suggest that overexpression of MLF can induce expression of the *cwp1-3* and *myb2* genes and cyst formation. The MLF-HA was detected in unique localization in some unknown cytosolic vesicles, named MLF vesicles (MVs), during vegetative growth and encystation using anti-HA antibody (Fig 1E, also see below). It was also detected at the edge of sucking disk in some cells during encystation (Fig 1E). Localization of MLF at the edge of sucking disk was also found previously [55].

### Knockdown of *mlf* gene using 3 strategies

**Fig 2:**
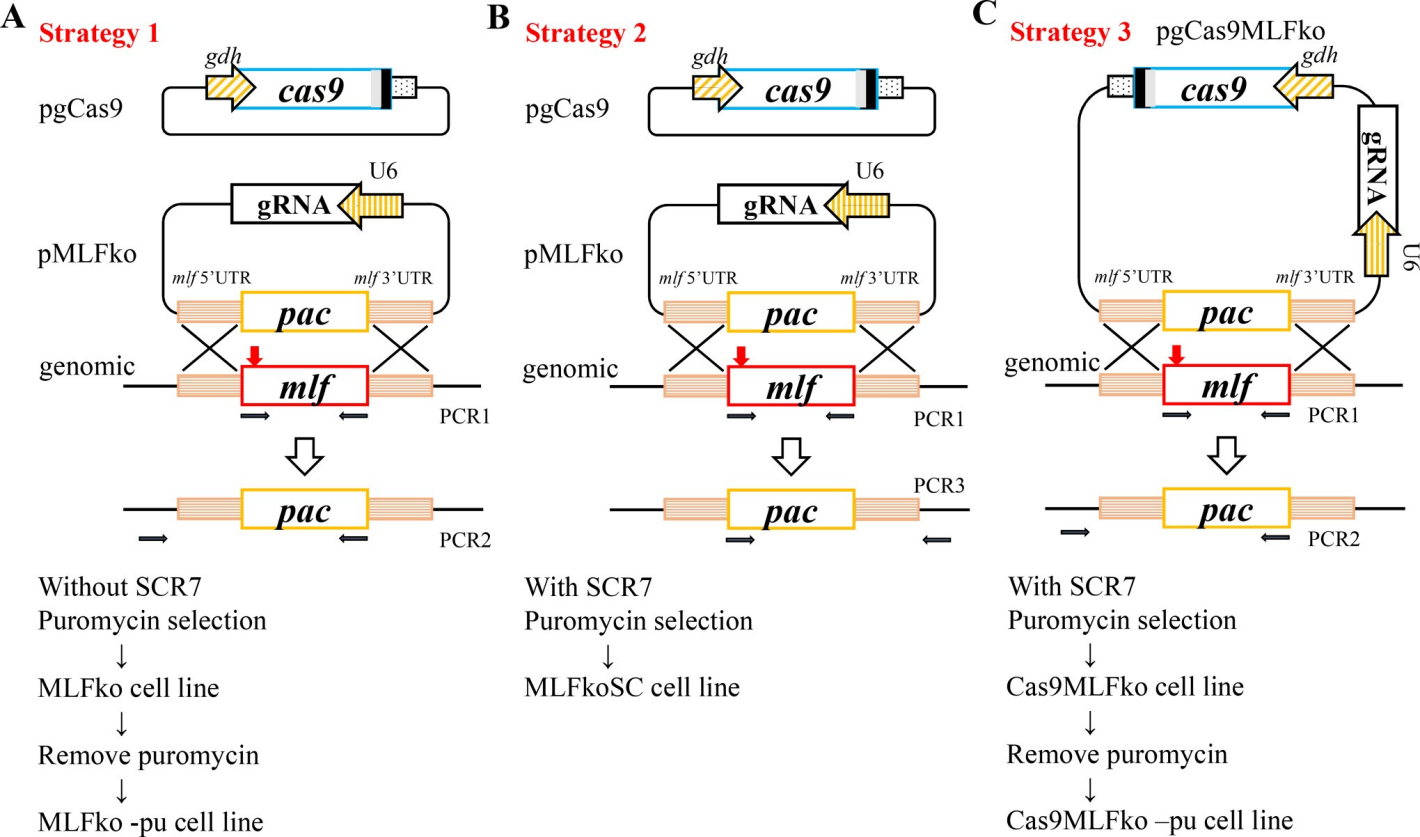
Strategies 1 to 3 for MLF knock out. (A) Strategy 1, diagrams of the pgCas9 and pMLFko plasmids. In construct pgCas9, the *cas9* gene is under the control of *gdh* promoter (striated box) and 3’ untranslated region of the *ran* gene (dotted box) and its product has a C-terminal nuclear localization signal (filled gray box) and an HA tag (filled black box). In construct pMLFko, a single gRNA is driven by the *Giardia* U6 promoter. The single gRNA includes a guide sequence targeting 20-nucleotides of the *mlf* gene (nt 115–134), which is located upstream three nucleotides of protospacer-adjacent motif (NGG sequence). pMLFko also has the HR template cassette which contains the 5’ and 3’ flanking region of the *mlf* gene as homologous arms and the *pac* selectable marker. The Cas9/gRNA cutting site in the genomic *mlf* gene is indicated by a red arrow. After introducing a double-stranded DNA break in the *mlf* gene, replacement of the genomic *mlf* gene with the *pac* gene will occur by HR. The pgCas9 and pMLFko constructs were transfected into *G*. *lamblia* WB trophozoites and MLFko stable transfectants were established under puromycin selection. The control cell line is trophozoites transfected with double amounts of 5’Δ5N-Pac plasmid and selected with puromycin. PCR1/2 were used for identification of knockout clones. Puromycin was removed from the MLFko and control cell lines to obtain the MLFko–pu and control–pu cell lines, respectively. (B) Strategy 2, diagrams of the pgCas9 and pMLFko plasmids, which are the same as in Fig 2A. These two constructs were transfected into trophozoites. An NHEJ inhibitor, SCR7, was added into the culture to increase HR. The MLFkoSC stable transfectants were established under puromycin selection. The control cell line is trophozoites transfected with double amounts of 5’Δ5N-Pac plasmid and selected with puromycin. (C) Strategy 3, diagrams of the pgCas9MLFko plasmid, which contains all the elements, including the expression cassettes of Cas9 and gRNA, and the HR template cassette which contains the 5’ and 3’ flanking region of the *mlf* gene as homologous arms and the *pac* selectable marker. After introducing a double-stranded DNA break in the *mlf* gene (red arrow), replacement of the genomic *mlf* gene with the *pac* gene will occur by HR. The pgCas9MLFko plasmid was transfected into trophozoites. An NHEJ inhibitor, SCR7, was added to increase HR. The Cas9MLFko stable transfectants were established under puromycin selection. The control cell line is trophozoites transfected with 5’Δ5N-Pac plasmid and selected with puromycin. In another experiment, puromycin was removed from the Cas9MLFko cell line to obtain the Cas9MLFko–pu cell line. The control cell line is wild type nontransfected WB trophozoites.

We combined the CRISPR/Cas9 system and the previously reported stable transfection system that was involved in maintenance of episomal plasmids under puromycin selection [20]. We constructed two plasmids, pgCas9 and pMLFko, that contains a Cas9 expression cassette, a gRNA expression cassette, and an HR template cassette (Fig 2A). These two constructs were transfected into *Giardia* trophozoites and MLFko stable transfectants were established under puromycin selection (Fig 2A). The pgCas9 plasmid was predicted to be present transiently in the absence of selection markers (Fig 2A)[56,57]. The pMLFko plasmid was predicted to be present stably in the presence of a *pac* selection marker (Fig 2A)[20]. PCR1 was used to detect the loss of *mlf* gene in genomic DNA (Fig 2A). The forward primer for PCR2 annealed to sequence upstream of the HR template of genomic DNA, but not inside the HR template, so PCR2 can detect the insertion of *pac* gene in genomic DNA (Fig 2A). We named this strategy as strategy 1. We also tried to use Scr7 to increase HR efficiency, named strategy 2 (Fig 2B). In strategy 3, an all-in-one plasmid system was established and tested (Fig 2C).

### Knockdown of *mlf* gene using a two-plasmid CRISPR/Cas9 system

**Fig 3:**
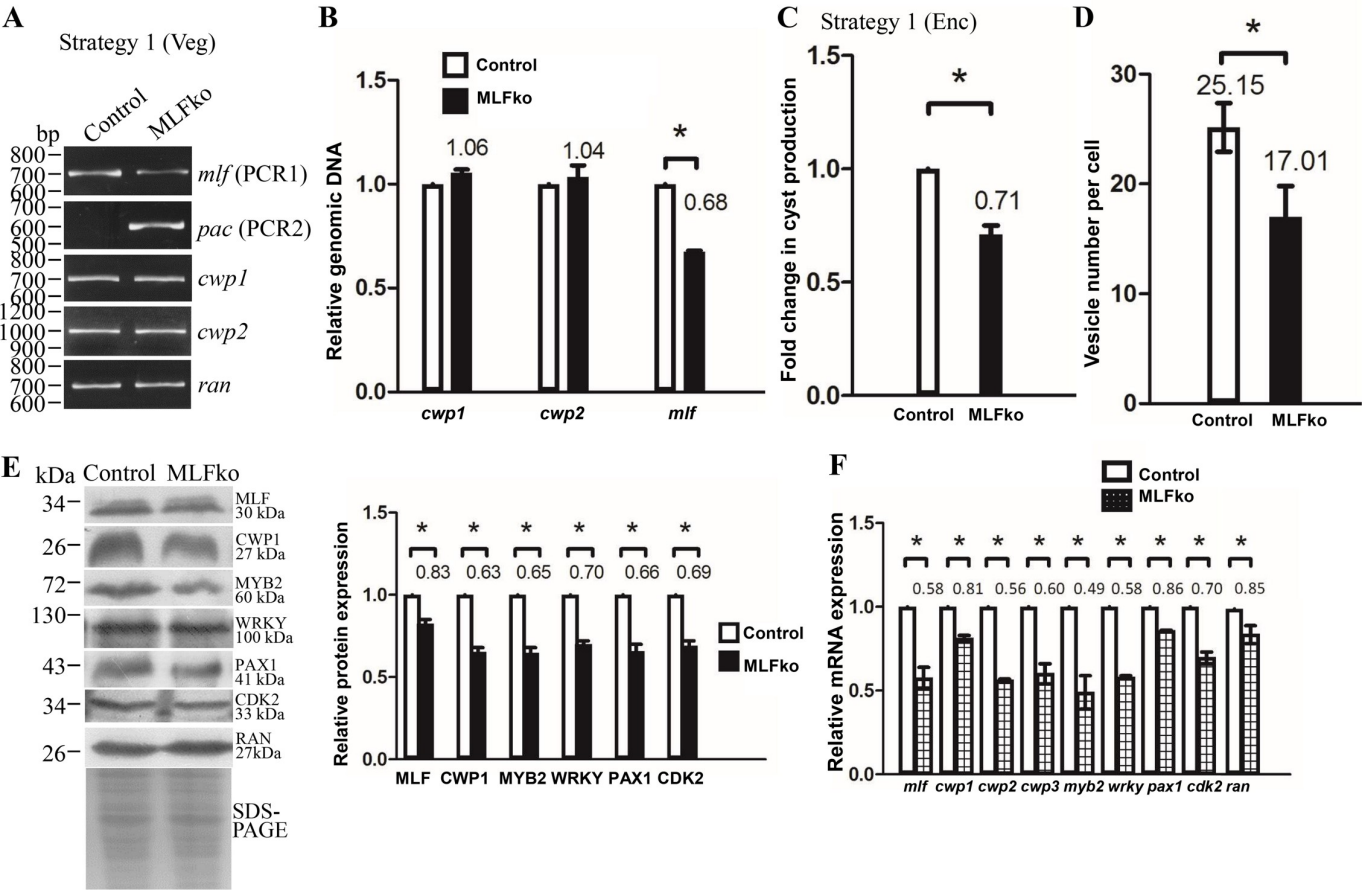
Decrease of *mlf*, *cwp1-3*, *myb2*, *wrky*, *pax1*, and *cdk2* gene expression by MLF knock down during encystation using strategy 1. (A) Partial replacement of the *mlf* gene with the *pac* gene in the MLFko cell line confirmed by PCR. Puromycin was kept in the MLFko and control cell lines as described in Fig 2A. Genomic DNA was isolated from MLFko and control cell lines cultured in growth medium (vegetative growth, Veg). PCR was performed using primers specific for *mlf* (PCR1), *pac* (PCR2), *cwp1*, *cwp2*, and *ran* genes, respectively. Products from the *cwp1*, *cwp2*, and *ran* genes are internal controls. (B) Partial disruption of *mlf* gene in the MLFko cell line confirmed by real-time PCR. Real-time PCR was performed using genomic DNA and primers specific for *mlf*, *cwp1*, *cwp2*, and *ran* genes, respectively. The *mlf*, *cwp1*, and *cwp2* DNA levels were normalized to the *ran* DNA level.–Fold changes in DNA levels are shown as the ratio of DNA levels in MLFko cell line relative to control cell line. Results are expressed as the means ± S. E. (error bars) of at least three separate experiments. *, P <0.05. (C) Cyst formation decreased by MLF knock down in the MLFko cell line during encystation. The control and MLFko cell lines were cultured in encystation medium and then subjected to cyst count as described under “Material and Methods” and Fig 1B. (D) Decrease of number of MVs by MLF knock down in the MLFko cell line. The control and MLFko cell lines were cultured in encystation medium and then subjected to immunofluorescence analysis using anti-MLF antibody for detection. MVs in MLFko and control cell lines were quantitated using Imaris software. MLF localized to fewer MVs in the MLFko cell line relative to the control cell line. *, P <0.05 (n = 30–70 cells/condition). (E) Knock down of *mlf* gene decreased the levels of CWP1, MYB2, and other proteins in the MLFko cell line. The control and MLFko cell lines were cultured in encystation medium and then subjected to SDS-PAGE and Western blot analysis as described in Fig 1C. The blot was probed with anti-MLF, anti-CWP1, anti-MYB2, anti-WRKY, anti-PAX1, anti-CDK2, and anti-RAN antibodies, respectively. The intensity of bands from three Western blot assays was quantified using Image J. The ratio of each target protein over the loading control RAN is calculated. Fold change is calculated as the ratio of the difference between MLFko cell line and the control cell line, to which a value of 1 was assigned. Results are expressed as mean ± SD. *P<0.05. (F) Decrease of multiple gene expression by MLF knock down in the MLFko cell line. The control and MLFko cell lines were cultured in encystation medium and then subjected to quantitative real-time RT-PCR analysis using primers specific for *mlf*, *cwp1*, *cwp2*, *cwp3*, *myb2*, *wrky*, *pax1*, *cdk2*, *ran*, and 18S ribosomal RNA genes, respectively. Transcript levels were normalized to 18S ribosomal RNA levels.–Fold changes in mRNA expression are shown as the ratio of transcript levels in the MLFko cell line relative to the control cell line. Results are expressed as the means ± S. E. of at least three separate experiments. *, P <0.05. The *ran* mRNAs slightly decreased.

We found a successful knockdown of the *mlf* gene, but not a complete knockout of the *mlf* gene using strategy 1 (Fig 3A and 3B). The replacement of the *mlf* gene with the *pac* gene in MLFko cell line was confirmed by PCR using PCR1 and PCR2 and sequencing analysis of genomic DNA (Figs 2A, 3A and S2 Fig). The results from PCR and quantitative real-time PCR show a successful disruption of the *mlf* gene by about 32% and a partial replacement of the *mlf* gene with the *pac* gene (Fig 3A and 3B). The level of cyst formation significantly decreased in the MLFko cell line relative to the control cell line during encystation (Fig 3C). MLF was detected in cytosolic vesicles, named MLF vesicles (MVs) (see above). Number of MVs also significantly decreased in the MLFko cell line relative to the control cell line (Fig 3D). Western blot analysis confirmed the decrease of the MLF protein level in the MLFko cell line relative to the control cell line during encystation (Fig 3E). The levels of the CWP1, MYB2, WRKY, PAX1, and CDK2 proteins also decreased in the MLFko cell line relative to the control cell line (Fig 3E). We further analyzed whether the transcript levels were changed by quantitative real-time analysis and RT-PCR, and found that the levels of *mlf*, *cwp1-3*, *myb2*, *wrky*, *pax1*, or *cdk2* mRNAs significantly decreased in the MLFko cell line relative to the control cell line (Fig 3F and S2 Fig). Similar results were obtained from the MLFko cell line during vegetative growth (S3 Fig). The results suggest a decrease of encystation related gene expression and cyst formation by knockdown of *mlf* gene using strategy 1.

**Fig 4:**
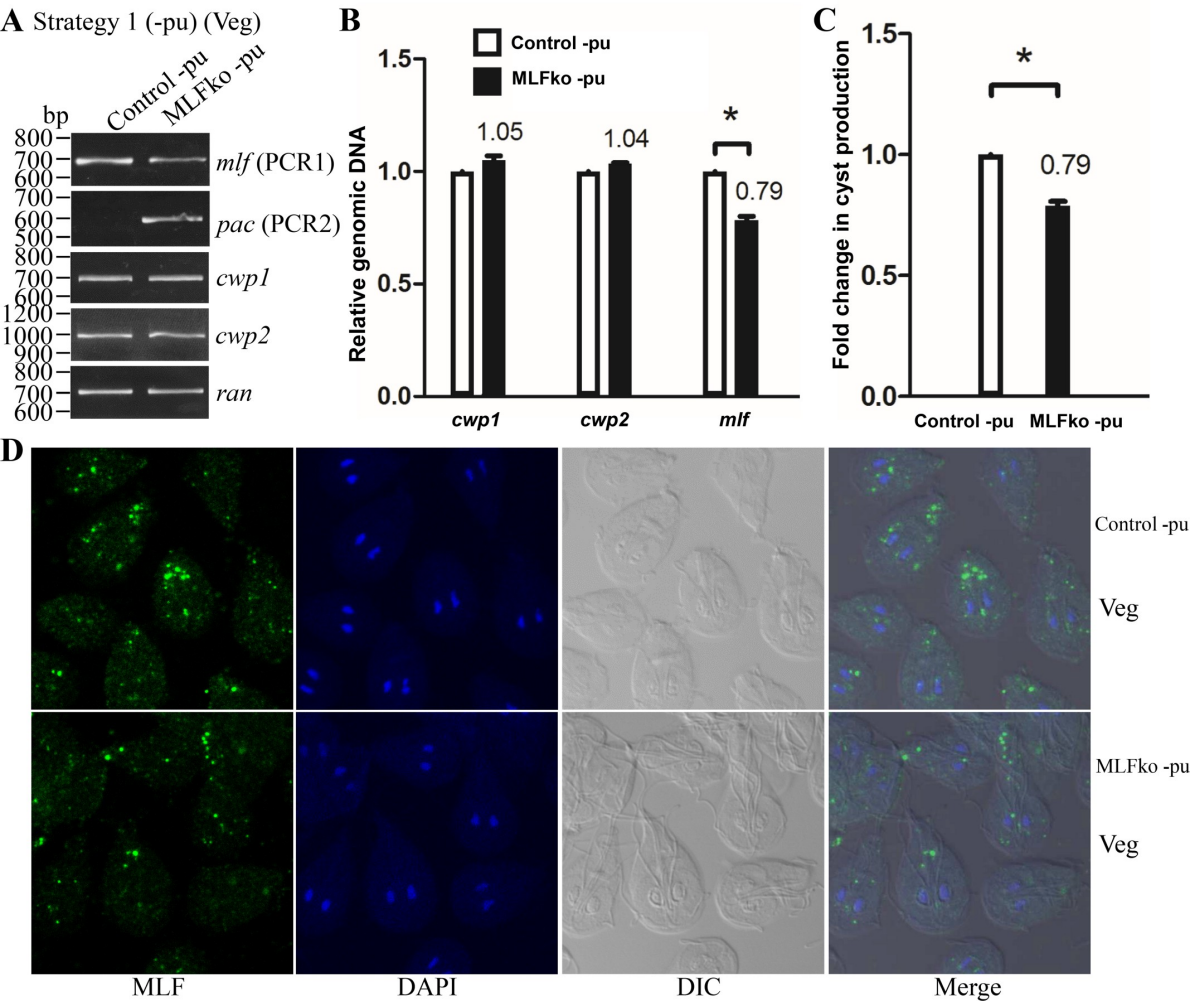
Decrease of cyst formation and number of MVs by MLF knock down after the removal of puromycin during vegetative growth using strategy 1. (A) Partial replacement of the *mlf* gene with the *pac* gene in the MLFko–pu cell line confirmed by PCR. Puromycin was removed from the MLFko and control cell lines to obtain the MLFko–pu and control–pu cell lines, respectively, as described in Fig 2A. Genomic DNA was isolated from MLFko–pu and control -pu cell lines cultured in growth medium (vegetative growth, Veg). PCR was performed using primers specific for *mlf* (PCR1), *pac* (PCR2), *cwp1*, *cwp2*, and *ran* genes, respectively, as described in Fig 3A. (B) Partial disruption of the *mlf* gene in the MLFko -pu cell line confirmed by real-time PCR. Real-time PCR was performed using genomic DNA and primers specific for *mlf*, *cwp1*, *cwp2*, and *ran* genes, respectively, as described in Fig 3B. (C) Cyst formation decreased by MLF knock down in the MLFko–pu cell line. The control–pu and MLFko–pu cell lines were cultured in growth medium and then subjected to cyst count as described under “Material and Methods” and Fig 1B. (D) Decrease of number of MVs by MLF knock down in the MLFko–pu cell line. The control–pu and MLFko–pu cell lines were cultured in growth medium and then subjected to immunofluorescence analysis using anti-MLF antibody for detection. MLF localized to fewer MVs in the MLFko–pu cell line relative to the control–pu cell line. The left panel shows that the MLF protein is localized to the vesicles in the cytoplasm. The middle panels show the DAPI staining of cell nuclei and differential interference contrast images. The right panel shows the merged images.

It is better to analyze results without puromycin, as puromycin may affect gene expression [48]. After selection, we further removed puromycin from the MLFko cell line to obtain the MLFko–pu cell line. The replacement of the *mlf* gene with the *pac* gene in MLFko–pu cell line was confirmed by PCR (Fig 4A). The results from PCR and quantitative real-time PCR show a successful disruption of the *mlf* gene by about 21% and a partial replacement of the *mlf* gene with the *pac* gene (Fig 4A and 4B). The level of cyst formation significantly decreased in the MLFko–pu cell line relative to the control–pu cell line (Fig 4C). MLF was detected in MVs by immunofluorescence assays with the anti-MLF antibody (Fig 4D). Number of MVs also significantly decreased in the MLFko–pu cell line relative to the control–pu cell line (Fig 4D and S4 Fig). Western blot analysis confirmed the decrease of the MLF protein level in the MLFko–pu cell line relative to the control–pu cell line during vegetative growth (S4 Fig). The levels of the CWP1, MYB2, WRKY, PAX1, and CDK2 proteins also obviously decreased in the MLFko–pu cell line relative to the control–pu cell line (S4 Fig). We further analyzed whether the transcript levels were changed by quantitative real-time analysis, and found that the levels of *mlf*, *cwp1-3*, *myb2*, *wrky*, *pax1*, or *cdk2* mRNAs significantly decreased in the MLFko–pu cell line relative to the control–pu cell line (S4 Fig). Similar results were obtained from the MLFko–pu cell line during encystation (S5 Fig). The results suggest a decrease of encystation related gene expression and cyst formation by knockdown of *mlf* gene using strategy 1 without puromycin.

### Scr7 increased gene disruption efficiency

We further tried to increase HR as a repair pathway for knocking in of the *pac* gene and for replacement of the *mlf* gene using strategy 2, which is similar to strategy 1, except that an NHEJ inhibitor, Scr7, was added (Fig 2B)[40]. Addition of Scr7 significantly decreased *Giardia* growth (S6 Fig). We transfected *Giardia* trophozoites with the same two constructs as described above (Fig 2B). After transfection, we added puromycin for selection and also Scr7 in the first replenishment. MLFkoSC stable transfectants were further established under puromycin selection.

The replacement of the *mlf* gene with the *pac* gene in MLFkoSC cell line was confirmed by PCR using PCR1 and PCR3 and sequencing analysis of genomic DNA (S2 and S6 Figs). The results from PCR and quantitative real-time PCR show a successful disruption of the *mlf* gene by about 53% and a partial replacement of the *mlf* gene with the *pac* gene (S6 Fig). The level of cyst formation significantly decreased in the MLFkoSC cell line relative to the control cell line (S6 Fig). Number of MVs also significantly decreased in the MLFkoSC cell line relative to the control cell line (S6 Fig). Western blot analysis confirmed the decrease of the MLF protein level in the MLFkoSC cell line relative to the control cell line during vegetative growth (S6 Fig). The levels of the CWP1, MYB2, WRKY, PAX1, and CDK2 proteins also obviously decreased in the MLFkoSC cell line relative to the control cell line (S6 Fig). We further analyzed whether the transcript levels were changed by quantitative real-time analysis, and found that the levels of *mlf*, *cwp1-3*, *myb2*, *wrky*, *pax1*, or *cdk2* mRNAs significantly decreased in the MLFkoSC cell line relative to the control cell line (S6 Fig). Similar results were obtained from the MLFkoSC cell line during encystation (S7 Fig). The results suggest a decrease of encystation related gene expression and cyst formation by knockdown of *mlf* gene using strategy 2.

We also did No-Cas9 control and found that the *mlf* gene was not mutated (S8 Fig), suggesting that homologous recombination did not occur in the absence of Cas9 expression, even in the presence of gRNA and HR template (S8 Fig). We also did a No-HR-template control and found that the *mlf* gene was not mutated, suggesting that targeting the *mlf* gene using puromycin selection has more efficiency as compared to strategy 2 (S6 and S8 Figs). The results from PCR and quantitative real-time PCR suggest that the *mlf* gene was not disrupted and that no replacement of the *pac* gene was found in the No-Cas9 control and No-HR-template control (S8 Fig). The levels of the MLF and CWP1 proteins did not change in the No-Cas9 control and No-HR-template control (S8 Fig).

**Fig 5:**
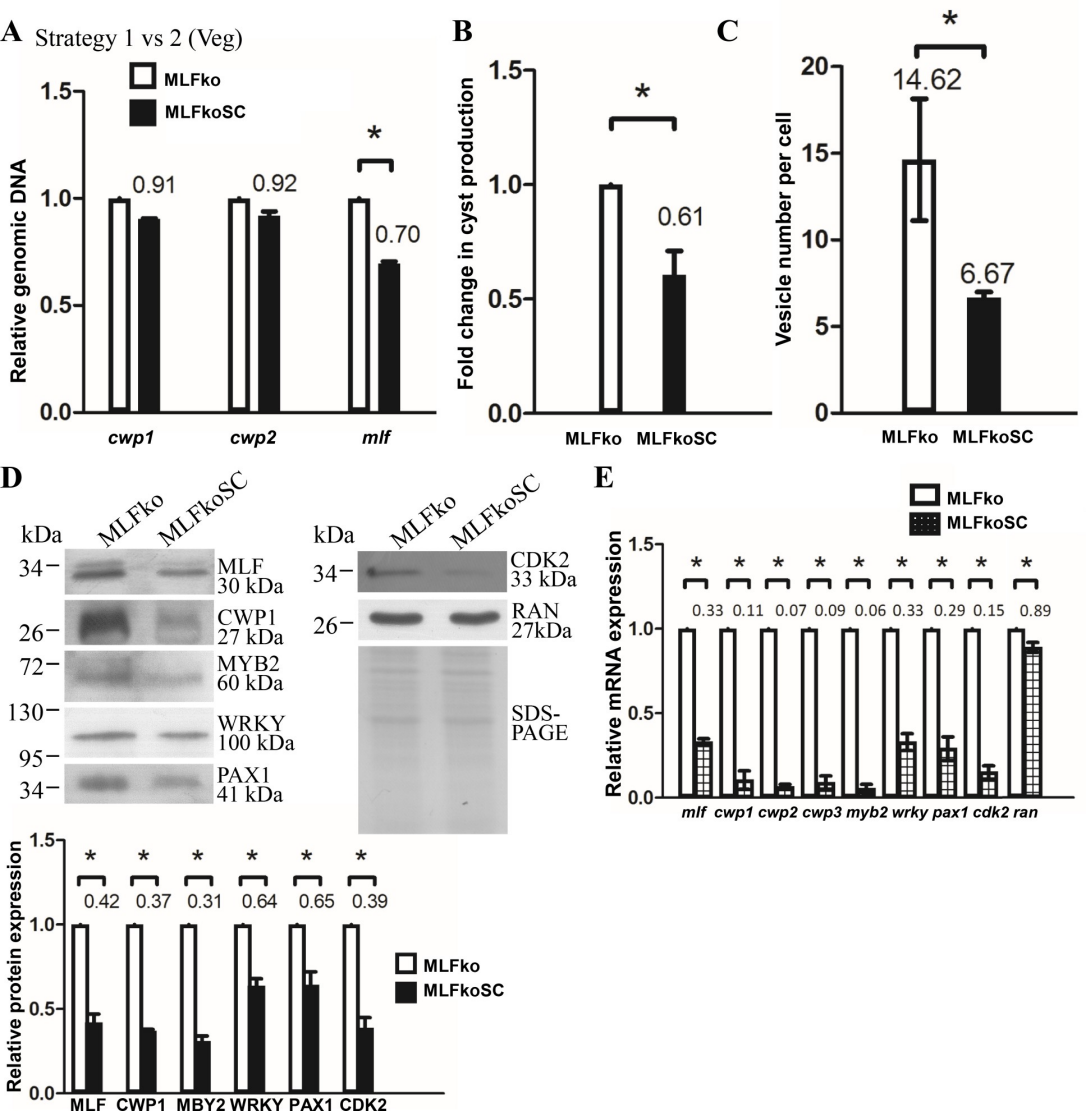
Increase of MLF knock down efficiency during vegetative growth using strategy 2 compared to strategy 1. (A) Increased disruption of the *mlf* gene in the MLFkoSC cell line relative to the MLFko cell line confirmed by real-time PCR. Genomic DNA was isolated from MLFko and MLFkoSC cell lines cultured in growth medium (vegetative growth, Veg). Real-time PCR was performed using primers specific for *mlf*, *cwp1*, *cwp2*, and *ran* genes, respectively, as described in Fig 3B. (B) MLF knock down decreased cyst formation in the MLFkoSC cell line relative to the MLFko cell line. The MLFko and MLFkoSC cell lines were cultured in growth medium and then subjected to cyst count as described under “Material and Methods” and Fig 1B. (C) Decrease of number of MVs by MLF knock down in the MLFkoSC cell line relative to the MLFko cell line. The MLFko and MLFkoSC cell lines were cultured in growth medium and then subjected to immunofluorescence analysis using anti-MLF antibody for detection as described in Fig 3D. (D) Knock down of *mlf* gene decreased the levels of CWP1, MYB2, and other proteins in MLFkoSC cell line relative to the MLFko cell line. The MLFko and MLFkoSC cell lines were cultured in growth medium and then subjected to SDS-PAGE and Western blot analysis as described in Fig 1C. The blot was probed with anti-MLF, anti-CWP1, anti-MYB2, anti-WRKY, anti-PAX1, anti-CDK2, and anti-RAN antibodies, respectively. The intensity of bands from three Western blot assays was quantified using Image J. The ratio of each target protein over the loading control RAN is calculated. Fold change is calculated as the ratio of the difference between MLFkoSC cell line and the MLFko cell line, to which a value of 1 was assigned. Results are expressed as mean ± SD. *P<0.05. (E) Decrease of multiple gene expression by MLF knock down in MLFkoSC cell line relative to the MLFko cell line. The MLFko and MLFkoSC cell lines were cultured in growth medium and then subjected to quantitative real-time RT-PCR analysis using primers specific for *mlf*, *cwp1*, *cwp2*, *cwp3*, *myb2*, *wrky*, *pax1*, *cdk2*, *ran*, and 18S ribosomal RNA genes, respectively, as described in Fig 3F.

To compare strategies 1 and 2, we further analyzed gene disruption efficiency in the MLFko and MLFkoSC cell lines. The replacement of the *mlf* gene with the *pac* gene in these cell lines was analyzed by quantitative real-time PCR of genomic DNA (Fig 5A). Addition of Scr7 increased gene disruption efficiency by about 30% (Fig 5A). The level of cyst formation significantly decreased in the MLFkoSC cell line relative to the MLFko cell line (Fig 5B). Number of MVs also significantly decreased in the MLFkoSC cell line relative to the MLFko cell line (Fig 5C). Western blot analysis confirmed the decrease of the MLF protein level in the MLFkoSC cell line relative to the MLFko cell line during vegetative growth (Fig 5D). The levels of the CWP1, MYB2, WRKY, PAX1, and CDK2 proteins also significantly decreased in the MLFkoSC cell line relative to the MLFko cell line (Fig 5D). We further analyzed whether the transcript levels were changed by quantitative real-time analysis, and found that the levels of *mlf*, *cwp1-3*, *myb2*, *wrky*, *pax1*, or *cdk2* mRNAs significantly decreased in the MLFkoSC cell line relative to the MLFko cell line (Fig 5E). Similar results were obtained during encystation (S9 Fig). We also found that the cyst wall is thinner in the MLFkoSC cell line relative to the MLFko cell line and that the cyst wall is thinner in the MLFko cell line relative to the control cell line (S9 Fig), correlating with the relative expression levels of *cwp1-3* genes (S3 and S7 Figs). The results suggest an increase of gene disruption efficiency by Scr7 treatment.

### Incorporation of all cassettes into one plasmid increased gene disruption efficiency

**Fig 6:**
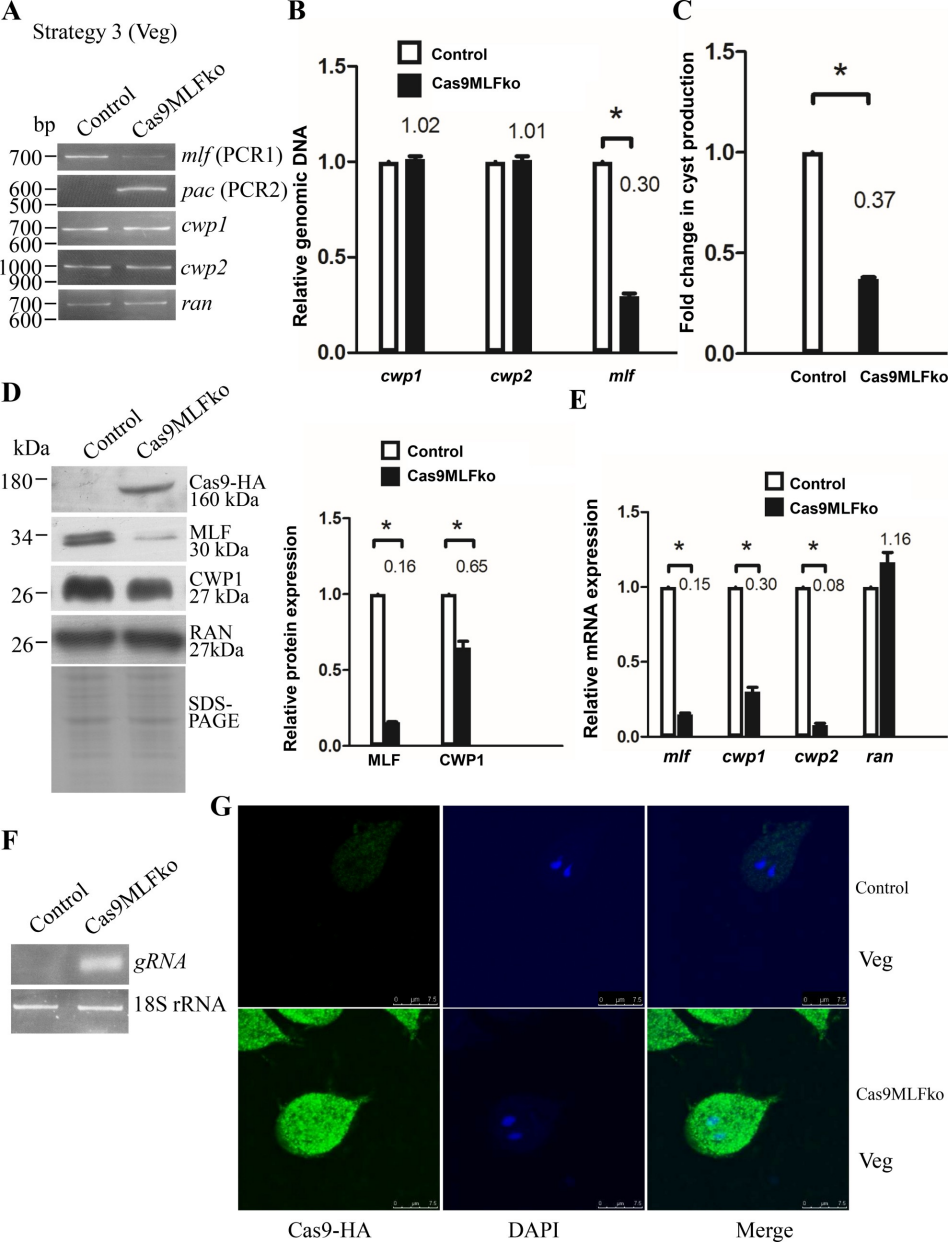
Decrease of *cwp1*, *cwp2*, and *myb2* gene expression by MLF knock down during vegetative growth using strategy 3. (A) Partial replacement of the *mlf* gene with the *pac* gene in the Cas9MLFko cell line confirmed by PCR. The pgCas9MLFko plasmid was transfected into trophozoites as described in Fig 2C. Genomic DNA was isolated from Cas9MLFko and control cell lines cultured in growth medium (vegetative growth, Veg). PCR was performed using primers specific for *mlf* (PCR1), *pac* (PCR2), *cwp1*, *cwp2*, and *ran* genes, respectively, as described in Fig 3A. (B) Partial disruption of the *mlf* gene in the Cas9MLFko cell line confirmed by real-time PCR. Real-time PCR was performed using genomic DNA and primers specific for *mlf*, *cwp1*, *cwp2*, and *ran* genes, respectively, as described in Fig 3A. (C) Cyst formation decreased by MLF knock down in the Cas9MLFko cell line. The control and Cas9MLFko cell lines were cultured in growth medium and then subjected to cyst count as described under “Material and Methods” and Fig 1B. (D) Knock down of *mlf* gene decreased the levels of MLF and CWP1 proteins in the Cas9MLFko cell line. The control and Cas9MLFko cell lines were cultured in growth medium and then subjected to SDS-PAGE and Western blot analysis as described in Fig 1C. The blot was probed with anti-HA, anti-MLF, anti-CWP1, and anti-RAN antibodies, respectively. The intensity of bands from three Western blot assays was quantified using Image J. The ratio of each target protein over the loading control RAN is calculated. Fold change is calculated as the ratio of the difference between Cas9MLFko cell line and the control cell line, to which a value of 1 was assigned. Results are expressed as mean ± SD. *P<0.05. (E) Decrease of *mlf*, *cwp1*, and *cwp2* gene expression by MLF knock down in the Cas9MLFko cell line. The control and Cas9MLFko cell lines were cultured in growth medium and then subjected to quantitative real-time RT-PCR analysis using primers specific for *mlf*, *cwp1*, *cwp2*, *ran*, and 18S ribosomal RNA genes, respectively, as described in Fig 3F. (F) RT-PCR analysis of gRNA expression in the in the Cas9MLFko cell line. The control and Cas9MLFko cell lines were cultured in growth medium and then subjected to RT-PCR analysis using primers specific for gRNA, and 18S ribosomal RNA genes, respectively. Similar levels of the 18S ribosomal RNA were detected. (G) Localization of Cas9-HA to the nuclei and cytoplasm in the Cas9MLFko cell line. The control and Cas9MLFko cell lines were cultured in growth medium and then subjected to immunofluorescence analysis using anti-HA antibody for detection. Cas9-HA localized to the nuclei and cytoplasm in the Cas9MLFko cell line but no signal was detected in the control cell line. The left panel shows that the Cas9-HA protein is localized to the nuclei and cytoplasm, but no signal was detected in the control cell line. The middle panel shows the DAPI staining of cell nuclei. The right panel shows the merged images.

We further tried to incorporate all three cassettes, including an HR template cassette, a Cas9 expression cassette, and a gRNA expression cassette, into one plasmid (Figs 2C and 6, strategy 3). After transfection of the pgCas9MLFko plasmid, we successfully obtained puromycin resistant Cas9MLFko transfectants, which are predicted to stably express Cas9 and a gRNA targeting the *mlf* gene (Figs 2C and 6).

The replacement of the *mlf* gene with the *pac* gene in Cas9MLFko cell line was confirmed by PCR using PCR1 and PCR2 and sequencing analysis of genomic DNA (S2 Fig and Fig 6A). The results from PCR and quantitative real-time PCR show a successful disruption of the *mlf* gene by about 70% and a partial replacement of the *mlf* gene with the *pac* gene (Fig 6A and 6B). The level of cyst formation significantly decreased in the Cas9MLFko cell line relative to the control cell line (Fig 6C). The levels of the MLF and CWP1 proteins and the levels of *mlf*, *cwp1*, or *cwp2* mRNAs significantly decreased in the Cas9MLFko cell line relative to the control cell line during vegetative growth (Fig 6D and 6E). We can detect the expression of HA tagged Cas9 protein in the Cas9MLFko cell line but not in the control cell line (Fig 6D). We also found that gRNA can be detected by RT-PCR analysis (Fig 6F). In addition, we found that the Cas9-HA protein localized to the nuclei and cytoplasm in the Cas9MLFko cell line, but no signal was detected in the control cell line (Fig 6G). The results suggest a decrease of encystation related gene expression and cyst formation by knockdown of *mlf* gene using strategy 3. Similar results were obtained from the Cas9MLFko cell line during encystation (S10 Fig).

We also obtained two clonal populations using single cell dilution method (S10 Fig). The results from PCR and quantitative real-time PCR show a successful disruption of the *mlf* gene by about 73% or 78% and a partial replacement of the *mlf* gene with the *pac* gene (S10 Fig).

We also did two gRNA controls using the same all-in one plasmid system with gRNA 1 or 2 (S11 Fig). The gRNA 1 targets a different region of the *mlf* gene (nt 61–80) as compared with strategy 1 (nt 115–134) (also see S1 Table). We found a successful disruption of the *mlf* gene by about 46% and a partial replacement of the *mlf* gene with the *pac* gene in gRNA control 1 (S11 Fig), suggesting that the second gRNA also worked. The levels of the MLF and CWP1 proteins obviously decrease in the gRNA control 1 (S11 Fig). The gRNA 2, which targets 20 mers of G, is a negative gRNA control (also see S1 Table). However, there was no replacement or mutation of the *mlf* gene in gRNA control 2 (S11 Fig), suggesting that the negative gRNA can not function in gene disruption. The levels of the MLF and CWP1 proteins did not change in the gRNA control 2 (S11 Fig).

We further tried to analyze results without puromycin. After selection, puromycin was removed from the Cas9MLFko cell line to obtain the Cas9MLFko -pu cell line (Fig 2C and S12 Fig). The control cell line is wild type nontransfected WB trophozoites. The results from quantitative real-time PCR show a successful disruption of the *mlf* gene by about 69% (S12 Fig).The level of cyst formation significantly decreased in the Cas9MLFko–pu cell line relative to the control cell line (S12 Fig). The levels of the MLF and CWP1 proteins and the levels of *mlf*, *cwp1*, or *cwp2* mRNAs obviously decreased in the Cas9MLFko–pu cell line relative to the control cell line during vegetative growth (S12 Fig). We did not detect the expression of HA tagged Cas9 protein in the Cas9MLFko -pu cell line or the control -pu cell line, possibly due to the removal of puromycin and loss of the plasmid (S12 Fig). Similar results were obtained from the Cas9MLFko–pu cell line during encystation (S13 Fig). The results suggest a decrease of encystation related gene expression and cyst formation by knockdown of *mlf* gene using strategy 3 without puromycin.

## Discussion

**Fig 7:**
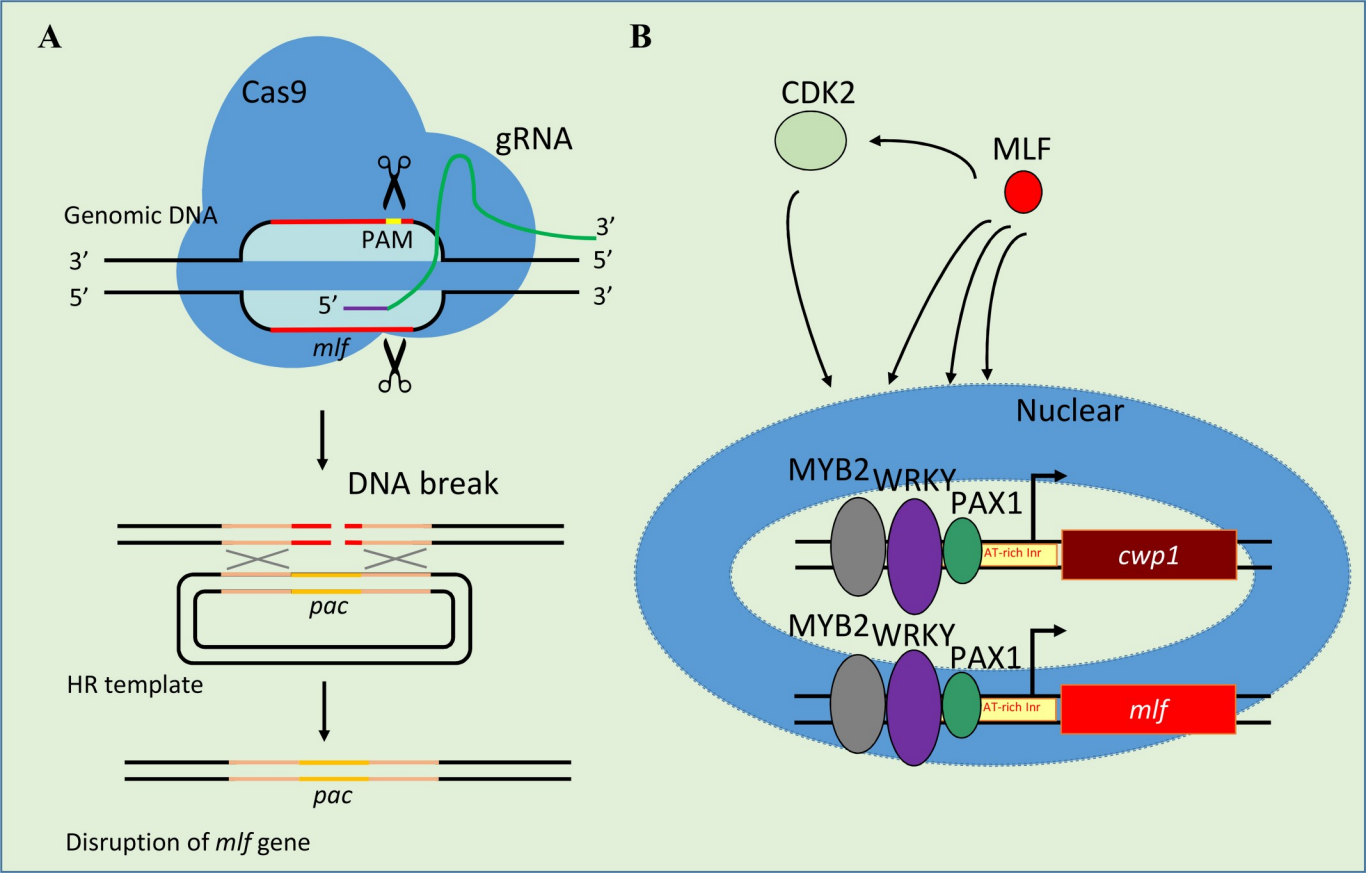
MLF increases and induces cyst formation during encystation. (A) Disruption of the *mlf* gene by CRISPR/Cas9 system. A single gRNA includes a guide sequence targets the 20-nucleotides of the *mlf* gene which is located upstream three nucleotides of protospacer-adjacent motif (PAM, NGG sequence). An HR template cassette contains the 5’ and 3’ flanking region of the *mlf* gene as homologous arms and the *pac* selectable marker. Cas9 can form a complex with gRNA to target DNA by recognizing PAM sequence. After cleavage of DNA by Cas9/gRNA complex, dsDNA break will form and then replacement of the genomic *mlf* gene with the *pac* gene will occur by HR. (B) Increase of encystation-induced pathway during encystation. The gene encoding key components of the cyst wall, *cwp1*, is up-regulated by MYB2, WRKY, and PAX1 transcription factors during encystation. These transcription factors can bind to AT-rich initiator (Inr) of the *cwp1* promoter. They can also activate *mlf* gene expression. The increase of MLF also can increase the levels of CWP1, MYB2, WRKY, PAX1, and CDK2. MLF was present in vegetative trophozoite stage at a lower level. During encystation, more MLF is produced by these transcription factors. MLF may further induce encystation by increasing this encystation-induced pathway in *Giardia*. The increase of transcription factors may further induce CWP1 and MLF expression, resulting in more cyst formation during encystation.

The protozoan *G*. *lamblia* that can form cysts to survive in the environment and to infect hosts, is a unique model diverging from the commonly studied eukaryotes. Studies in *Giardia* provide information for biology of the unicellular eukaryote, particularly of regulation of cell differentiation. Establishment and application of gene knockout systems are important works for studies of this organism. We have developed a CRISPR/Cas9 system to disrupt *mlf* gene using two plasmids to express Cas9 and gRNA (Figs 2A and 3, strategy 1). The disruption of *mlf* gene can be achieved by partial replacement of the *mlf* gene with the *pac* gene by HR (Fig 7A). To further improve the disruption efficiency, we added Scr7, an NHEJ inhibitor, to increase HR, and found an increase of the disruption efficiency on *mlf* gene from about 32% to about 53%, compared with the system without Scr7 (Figs 2B, 3B, and S6 Fig, strategy 2). Scr7 also worked in mammalian cells to increase knock-in efficiency via CRISPR/Cas9-coupled HR [39,40,41]. Canonical NHEJ works by DNA ligase IV and XRCC mediated joining of dsDNA break [40]. Alternative NHEJ works by DNA ligase I/III [40]. We only found one DNA ligase gene (open reading frame 7649) with low homology to ligase IV and III. Since Scr7 can inhibit DNA ligase IV, and also DNA ligase III [40,58], it is possible that Scr7 can target Giardia DNA ligase. This awaits future work to explore. A XRCC-like gene was found from *Giardia* genome database (open reading frame 6918) but also with very low homology. Scr7 may inhibit *Giardia* DNA repair since Scr7 can inhibit *Giardia* growth (S6 Fig). In addition, Scr7 can increase gene disruption efficiency in our system, suggesting that *Giardia* may have an unusual NHEJ pathway which still can be inhibited by Scr7. The importance of NHEJ for eukaryotic genome stability also suggests the presence of NHEJ in *Giardia*, which awaits further studies to clarify [59].

Transfection studies for gene expression have been used to investigate gene function in *Giardia*. A transient DNA transfection system employed a *gdh* promoter to drive a luciferase gene, which expressed transiently and only lasted for one day after transfection [56]. Stable transfection is more useful than transient transfection due to relatively long time and higher expression levels [19,20]. In our two-plasmid-CRISPR/Cas9 system, we used a transient transfection system to express the Cas9 protein, which was tagged with a C-terminal nuclear localization signal (Figs 2A and 3, strategy 1) [60]. We were not able to detect the expression of Cas9 protein by Western blot (Fig 3E), possibly due to low amounts of Cas9 expressed from the transient transfection system [19,56]. The successful results for MLF knockdown suggest that a little amount of the transiently expressed Cas9 is enough for its function after transfection [56]. We also tried to express Cas9 using stable transfection of an all-in-one plasmid in *Giardia* (Figs 2C and 6, strategy 3), although it has been reported that stably expressed Cas9 is toxic to *Toxoplasma* [61]. The all-in-one plasmid system was predicted to stably express Cas9 and a gRNA targeting the *mlf* gene, as we were able to detect the stably expressed Cas9 protein by Western blot and immunofluorescence (Fig 6D and 6G) and found an increase of disruption efficiency on *mlf* gene from about 53% to about 70% as compared with the two-plasmid system (Figs 2B and 7B, S6 Fig, strategy 3). We can detect the expressed Cas9 in both nuclei and cytoplasm in the all-in one plasmid system with the best gene disruption efficiency (Fig 6G), suggesting that partial localization of Cas9 to the nuclei is still functional. It was reported that Cas9 did not successfully enter nuclei using the HASKKKRKVAPKKKRKVDKKYSIGL sequence as a nuclear localization signal [22]. We used KKKRKV as the nuclear localization signal, which is shorter and can target tetracycline repressor to the nuclei in our tetracycline inducible system [60]. We tried to avoid the potential off-target effect by searching the NCBI Nucleotide database with our designed guide sequence. Reduction of Cas9 expression is also a way to reduce the off-target effect [62]. Our strategy 1 with transient expression of Cas9 provides a limited expression of Cas9 (see above).

In our two-plasmid system, we transfected a plasmid form of the HR template cassette that can be maintained by stable transfection using puromycin selection, allowing successfully introducing insertion of the *pac* drug resistance gene (Figs 2A, 3, and S6 Fig, strategy 1). We found a similar gene disruption efficiency on *mlf* gene and protein and mRNA expression whether the results were measured from the time immediately after selection or 1 month after selection. This suggests a relatively long and stable system. In the case of the plasmid form of the HR template with stable transfection, subsequent analysis was performed after removal of puromycin for a month to avoid the interference of plasmids and puromycin [19,48]. We found that the plasmid was not present after removal of puromycin for 1 month. Similarly, it has been found that the plasmid was not present after removal of G418 for 6 weeks [22].

Insertion of a whole plasmid, including the vector sequence to *Giardia* can be achieved by transfection of linear plasmid cut by restriction enzyme and drug selection [63]. In our study, gene replacement is achieved using circular plasmid and results in insertion of only the HR template fragment without the vector sequence (Fig 2A, strategy 1). We also found that the process is gRNA dependent (S11 Fig).

We did two gRNA controls with Cas9 and HR template in the all-in one system (S11 Fig). We found a successful disruption of the *mlf* gene in the gRNA control 1, which targets a different region of the *mlf* gene (nt 61–80) as compared with strategy 1 (nt 115–134) (also see S1 Table), suggesting that the second gRNA also worked. However, there was no mutation of the *mlf* gene in gRNA control 2 (S11 Fig), which has 20 mers of C as guide sequence targeting 20 mers of G, suggesting that the negative gRNA can not target the *mlf* genes. In addition, gRNA can be detected in the successful all-in-one system, suggesting that it was functional (Fig 6F). We also found that the process is Cas9 dependent (S8 Fig). There was no mutation of *mlf* gene in the No-Cas9 control (S8 Fig), which lacks Cas9 but has gRNA and HR template as compared with strategy 2 (Fig 2B). These results suggest that Cas9 is essential for the CRISPR/Cas9 system. We also found that the process is HR template dependent (S8 Fig). There was no mutation of the *mlf* gene in the No-HR-template control (S8 Fig), which lacks HR template, but has Cas9 and gRNA as compared with strategy 1 (Fig 2A), suggesting that targeting the *mlf* gene using puromycin selection has more efficiency. The presence of Cas9 and gRNA did not result in NHEJ, possibly due to low expression of transiently expressed Cas9 and gRNA (see above). We also performed single cell dilution for the all-in-one plasmid system and found that the obtained two clonal populations have a similar disruption efficiency of 73% and 78% as compared with their original cultures (70%) (Fig 6B and S10 Fig), suggesting homogeneity of the original population. A Cre/loxP system allows persistence of gene disruption in the absence of drug selection in *Giardia* [21,22]. It is interesting to compare the CRISPR/Cas9 and Cre-lox systems in *Giardia*.

Because of tetraploid genome and two nuclei, it could be hard to disrupt a gene completely in *Giardia*. To date, we can only knockdown *mlf* gene expression, but not complete knockout *mlf* gene. The best gene disruption efficiency we obtained is about 70% (Fig 6, strategy 3). Because puromycin had been added during selection, it is not possible to for the cells to preform HR by one more round of transfection and puromycin selection. The examples of successful knockout of target genes in other organisms suggest the potential of CRISPR/Cas9 to knockout genes in *Giardia* [28–32]. The potential of the CRISPR/Cas9 system to complete knockout genes of interest in *Giardia* awaits further studies to explore. Using a strong promoter to drive the expression of *cas9* gene could improve knockout efficiency. Our CRISPR/Cas9 system still provides a valuable tool for partial replacement of an essential gene for gene disruption in *Giardia* studies. It has been known that viability is low in *Drosophila* MLF null mutant embryo [64]. The success of MLF knockdown with our CRISPR/Cas9 system could be due to that MLF is a nonessential gene for *Giardia* growth. This is correlated with what we found that the major function of MLF is inducing encystation. To date, we do not know if the complete knockout of MLF may lead to death of *Giardia*. The confirmation that *mlf* gene is essential (death) or nonessential (growth) requires successful knockout of *mlf* gene. A complete knockout of an essential gene may lead to cell death, so it is hard to determine the gene function for that case. We have tried to knockdown 12 genes, including *mlf* by our CRISPR/Cas9 system, but only have obtained successful growth of 4 mutant cell lines after gene disruption under drug selection. We only generated the knockdown but not knockout mutants for these 4 genes. It is possible that the other 8 genes whose mutants cannot be obtained could be essential genes. Combining the CRISPR/Cas9 system and tetracycline inducible system can allow an adjustable knockdown of a target gene in the future [60,65].

Three genes encoding key components of the cyst wall, cyst wall proteins, are highly expressed during *Giardia* differentiation into dormant cysts [9–11]. It has been found that transcription factors MYB2, WRKY, and PAX1 can bind to specific promoter sequences and up-regulate the *cwp1-3* genes during encystation (Fig 7B)[13,15]. In addition, CDK2 is upstream of MYB2 by phosphorylation and activation of MYB2, leading to upregulation of *cwp1-3* genes during encystation [12]. The *myb2*, *wrky*, *pax1*, and *cdk2* genes are also all up-regulated during encystation [12–15]. Interestingly, during encystation, *mlf* gene is also up-regulated, suggesting a demand and a positive role of MLF in encystation [48]. We found that overexpression of MLF can induce the expression of *cwp1-3* and *myb2* genes and cyst formation (Fig 1). We developed a CRISPR/Cas9 system for *mlf* knockout studies in *Giardia*, but only obtained knockdown results. Knockdown of *mlf* gene led to a decrease of the MLF protein level and the number of MVs (Figs 3, 4, 5 and 6, and S9 Fig). Knockdown of *mlf* gene also led to a decrease in the levels of CWP1, MYB2, WRKY, PAX1, and CDK2 proteins and *cwp1-3*, *myb2*, *wrky*, *pax1*, and *cdk2* gene expression and cyst formation (Figs 3 and 5, and S9 Fig), suggesting a positive role of MLF in inducing *cwp1-3*, *myb2*, *wrky*, *pax1* and *cdk2* genes and cyst formation (Fig 7A and 7B). Because *Giardia* MLF localized to cytosolic MVs and it was not a nuclear protein, it is possible that MLF induced *cwp1-3* gene expression indirectly. MLF may upregulate MYB2, WRKY, PAX1 or CDK2, which have been known to induce *cwp1-3* gene expression [12–15], which then induces *cwp1-3* gene expression. Our knockdown studies suggest that regulation of CWP1, MYB2, WRKY, PAX1 or CDK2 protein levels by MLF could be critical for *Giardia* encystation. We also found that the *mlf* gene expression is activated by MYB2 and its gene promoter has the binding sites of MYB2 [13], suggesting a positive regulation cycle between MLF and MYB2. Human MLFs are important for normal hemopoietic differentiation and play a role in maintaining protein stability or in interacting with a secreted protein or a proteasome-regulatory system, but the specific role of human MLFs is not known [42– 47]. Role of MLF in *Giardia* MVs may be related to metabolism of unfolded mutant proteins, which await further studies to explore. Studies suggest that MLF is present in the membrane vesicles for mitosomes isolation [66,67]. However, detailed localization of MLF in mitosomes or some other vesicles awaits further study.

In this study, we have developed a CRISPR/Cas9 system in *G*. *lamblia*. This system could be used to disrupt a gene of interest for studying its roles in cell physiology and in cell differentiation, such as encystation or excystation. Such a system will also be useful to study the function of different proteins in a specific pathway *in vivo*. Our results also indicate that MLF can induce the expression of the *cwp* genes that are involved in differentiation in the primitive protozoan *G*. *lamblia*, suggesting that MLF may be functionally conserved and involved in cell differentiation. Our study leads to greater understanding of the evolution of eukaryotes during cell differentiation and help develop ways to interrupt the parasite life cycle.

## Supporting information

S1 Fig Sequence analysis of *Giardia* MLF. (A) Alignment of the MLF proteins. Specific sequence similarity search was performed against the GenBank database on NCBI's Web site using the BLASTP algorithm (http://blast.ncbi.nlm.nih.gov/Blast.cgi). This search identified similarity of *Giardia* MLF to *Drosophila* MLF human MLF1 and MLF2 in the GenBank database. Sequence of MLF proteins is analyzed by ClustalW 1.83, including *Drosophila* MLF human MLF1 and MLF2 (accession numbers are AFH08108.1, NP_071888.1, and NP_005430.1), and *Giardia* MLF (orf number 16424 in *Giardia* genome database). The gray boxes indicate the MLFIP domain of *Giardia* MLF predicted by pfam (http://pfam.sanger.ac.uk/). (B) Schematic representation of the *Giardia* MLF protein. The gray box indicates the MLFIP domain, as predicted by pfam. The residue numbers are shown.
(PDF)

S2 Fig RT-PCR analysis and sequence of PCR2 product from strategy 1. (A) RT-PCR analysis of gene expression in the MLF-overexpressing cell line. The 5’Δ5N-Pac and pPMLF stable transfectants were cultured in growth medium and then subjected to RT-PCR analysis. PCR was performed using primers specific for *mlf-ha*, *mlf*, *cwp1*, *cwp2*, *cwp3*, *myb2*, *ran*, and 18S ribosomal RNA genes, respectively. Similar levels of the *ran* mRNAs and 18S ribosomal RNA were detected. (B) Overexpression of MLF increased the levels of MLF proteins. The 5’Δ5N-Pac and pPMLF stable transfectants were cultured in growth medium and then subjected to SDS-PAGE and Western blot analysis. The blot was probed with anti-MLF antibody. The result is the same as in Fig 1C, but the whole gel is shown. (C) Replacement of the *mlf* gene with the *pac* gene in the MLFko cell line confirmed by PCR2 and sequencing. Genomic DNA was isolated from MLFko and control cell lines cultured in growth medium. PCR was performed using primers specific for *pac* (PCR2 in Fig 2A), which are PCR2F for bold region 1 and PCR2R for bold region 2, to verify the integration of *pac* gene into the correct region in genomic DNA. The sequence results obtained from the PCR2 product are shown as underlined letters. Capital letters indicate the coding sequence for *pac* gene, which starts at ATG and stops at TGA. This indicates the replacement of the *mlf* gene with the *pac* gene. The region used to clone the *mlf* 5’ region into the pMLFko plasmid for HR is shown in red, which is also between the sequence of MLF 5HF and MLF 5NR. The underlined and lower case letters, which are upstream and outside of the red region of MLF 5HF and MLF5NR, indicate that HR occurred in the sequence of *mlf* 5’ region and that the *pac* gene was integrated in the genomic DNA. Replacement of the *mlf* gene with the *pac* gene in the MLFkoSC and Cas9MLFko cell line was also confirmed by PCR2 and sequencing with the same sequencing results. (D) Replacement of the *mlf* gene with the *pac* gene in the MLFko cell line confirmed by PCR3 and sequencing. Genomic DNA was isolated from MLFko and control cell lines cultured in growth medium. PCR was performed using primers specific for *pac* (PCR3), which are PCR3F for bold region 1 and PCR3R for bold region 2, to verify the integration of *pac* gene into the correct region in genomic DNA. The sequence results obtained from the PCR3 product are shown as underlined letters. Capital letters indicate the coding sequence for *pac* gene, which starts at ATG and stops at TGA. This indicates the replacement of the *mlf* gene with the *pac* gene. The region used to clone the *mlf* 3’ region into the pMLFko plasmid for HR is shown in red, which is also between the sequence of MLF3XF and MLF3KR. The underlined and lower case letters, which are upstream and outside of the red region of MLF3XF and MLF3KR, indicate that HR occurred in the sequence of *mlf* 3’ region and that the *pac* gene was integrated in the genomic DNA. Replacement of the *mlf* gene with the *pac* gene in the MLFkoSC and Cas9MLFko cell line was also confirmed by PCR3 and sequencing with the same sequencing results. (E) RT-PCR analysis of gene expression in the MLFko cell line during encystation. The control and MLFko cell lines were cultured in encystation medium and then subjected to RT-PCR analysis using primers specific for *mlf*, *cwp1*, *cwp2*, *cwp3*, *myb2*, *wrky*, *pax1*, *cdk2*, *ran*, and 18S ribosomal RNA genes, respectively. Similar levels of the 18S ribosomal RNA were detected. The *ran* mRNAs slightly decreased.
(PDF)

S3 Fig Decrease of *mlf*, *cwp1-3*, *myb2*, *wrky*, *pax1*, and *cdk2* gene expression by MLF knock down during vegetative growth using strategy 1. (A) Cyst formation decreased by MLF knock down in the MLFko cell line during vegetative growth. The control and MLFko cell lines were cultured in growth medium for 24h (Enc) and then subjected to cyst count as described under “Materials and Methods” and Fig 1B. (B) Decrease of number of MVs by MLF knock down in the MLFko cell line during vegetative growth. The control and MLFko cell lines were cultured in growth medium and then subjected to immunofluorescence analysis using anti-MLF antibody for detection as described in Fig 3D. (C) Knock down of *mlf* gene decreased the levels of CWP1, MYB2, and other proteins in the MLFko cell line during vegetative growth. The control and MLFko cell lines were cultured in growth medium and then subjected to SDS-PAGE and Western blot analysis, as described in Fig 1C. The blot was probed with anti-HA, anti-MLF, anti-CWP1, anti-MYB2, anti-WRKY, anti-PAX1, anti-CDK2, and anti-RAN antibodies, respectively. The intensity of bands from three Western blot assays was quantified using Image J. The ratio of each target protein over the loading control RAN is calculated. Fold change is calculated as the ratio of the difference between MLFko cell line and the control cell line, to which a value of 1 was assigned. Results are expressed as mean ± SD. *P<0.05. (D) Decrease of multiple gene expression by MLF knock down in the MLFko cell line during vegetative growth. The control and MLFko cell lines were cultured in growth medium and then subjected to quantitative real-time RT-PCR analysis using primers specific for *mlf*, *cwp1*, *cwp2*, *cwp3*, *myb2*, *wrky*, *pax1*, *cdk2*, *ran*, and 18S ribosomal RNA genes, respectively, as described in Fig 3F. (E) RT-PCR analysis of gene expression in the MLFko cell line during vegetative growth. The control and MLFko cell lines were cultured in growth medium and then subjected to RT-PCR analysis using primers specific for *mlf*, *cwp1*, *cwp2*, *cwp3*, *myb2*, *wrky*, *pax1*, *cdk2*, *ran*, and 18S ribosomal RNA genes, respectively.
(PDF)

S4 Fig Decrease of *mlf*, *cwp1-3*, *myb2*, *wrky*, *pax1*, and *cdk2* gene expression by MLF knock down after the removal of puromycin during vegetative growth using strategy 1. (A) Quantification of MVs in MLFko–pu and control–pu cell lines using Imaris software. *, P <0.05 (n = 200–300 cells/condition). (B) Knock down of *mlf* gene decreased the levels of CWP1, MYB2, and other proteins in the MLFko–pu cell line. The control–pu and MLFko–pu cell lines were cultured in growth medium and then subjected to SDS-PAGE and Western blot analysis, as described in Fig 1C. The blot was probed with anti-MLF, anti-CWP1, anti-MYB2, anti-WRKY, anti-PAX1, anti-CDK2, and anti-RAN antibodies, respectively. (C) Decrease of multiple gene expression by MLF knock down in the MLFko–pu cell line. The control–pu and MLFko–pu cell lines were cultured in growth medium and then subjected to quantitative real-time RT-PCR analysis using primers specific for *mlf*, *cwp1*, *cwp2*, *cwp3*, *myb2*, *wrky*, *pax1*, *cdk2*, *ran*, and 18S ribosomal RNA genes, respectively, as described in Fig 3F.
(PDF)

S5 Fig Decrease of *mlf*, *cwp1-3*, *myb2*, *wrky*, *pax1*, and *cdk2* gene expression by MLF knock down after the removal of puromycin during encystation using strategy 1. (A) Cyst formation decreased by MLF knock down in the MLFko–pu cell line during encystation. The control–pu and MLFko–pu cell lines were cultured in encystation medium for 24h (Enc) and then subjected to cyst count as described under “Methods” and Fig 1B. (B) Quantification of MVs in MLFko–pu and control–pu cell lines during encystation. The control–pu and MLFko–pu cell lines were cultured in encystation medium and then subjected to immunofluorescence analysis using anti-MLF antibody for detection as described in Fig 3D. (C) Decrease of number of MVs by MLF knock down in the MLFko–pu cell line during encystation. The control–pu and MLFko–pu cell lines were cultured in encystation medium and then subjected to immunofluorescence analysis using anti-MLF antibody for detection as described in Fig 4D. (D) Knock down of *mlf* gene decreased the levels of CWP1, MYB2, and other proteins in the MLFko–pu cell line during encystation. The control–pu and MLFko–pu cell lines were cultured in encystation medium and then subjected to SDS-PAGE and Western blot analysis, as described in Fig 1C. The blot was probed with anti-MLF, anti-CWP1, anti-MYB2, anti-WRKY, anti-PAX1, anti-CDK2, and anti-RAN antibodies, respectively. The intensity of bands from three Western blot assays was quantified using Image J. The ratio of each target protein over the loading control RAN is calculated. Fold change is calculated as the ratio of the difference between MLFko–pu cell line and the control–pu cell line, to which a value of 1 was assigned. Results are expressed as mean ± SD. *P<0.05. (E) Decrease of multiple gene expression by MLF knock down in the MLFko–pu cell line during encystation. The control–pu and MLFko–pu cell lines were cultured in encystation medium and then subjected to quantitative real-time RT-PCR analysis using primers specific for *mlf*, *cwp1*, *cwp2*, *cwp3*, *myb2*, *wrky*, *pax1*, *cdk2*, *ran*, and 18S ribosomal RNA genes, respectively, as described in Fig 3F.
(PDF)

S6 Fig Decrease of *mlf*, *cwp1-3*, *myb2*, *wrky*, *pax1*, and *cdk2* expression by MLF knock down during vegetative growth using strategy 2. (A) Treatment with Scr7 decreased cell growth. The wild-type non-transfected WB cells were subcultured at an initial density of 1×10^6^ cells/ml in growth medium containing 33μM Scr7 for 24 h and then subjected to cell count. An equal volume of Me2SO was added to cultures as a negative control. The sum of total cells is expressed as a relative level over control. Values are shown as means ± S.E. of three independent experiments. *, P <0.05. (B) Partial replacement of the *mlf* gene with the *pac* gene in the MLFkoSC cell line confirmed by PCR. The pgCas9 and pMLFko constructs were transfected into trophozoites as described in Fig 2B. Puromycin was kept in the MLFkoSC and control cell lines. Genomic DNA was isolated from MLFkoSC and control cell lines cultured in growth medium (vegetative growth, Veg). PCR was performed using genomic DNA and primers specific for *mlf* (PCR1), *pac* (PCR3), *cwp1*, *cwp2*, and *ran* genes, respectively, as described in Fig 3A. (C) Partial disruption of the *mlf* gene in the MLFkoSC cell line confirmed by real-time PCR. Real-time PCR was performed using genomic DNA and primers specific for *mlf*, *cwp1*, *cwp2*, and *ran* genes, respectively, as described in Fig 3B. (D) MLF knock down decreased cyst formation in the MLFkoSC cell line during vegetative growth. The control and MLFkoSC cell lines were cultured in growth medium and then subjected to cyst count as described under “Methods” and Fig 1B. (E) Decrease of number of MVs by MLF knock down in the MLFkoSC cell line during vegetative growth. The control and MLFkoSC cell lines were cultured in growth medium and then subjected to immunofluorescence analysis using anti-MLF antibody for detection as described in Fig 3D. (F) Knock down of *mlf* gene decreased the levels of CWP1, MYB2, and other proteins in the MLFkoSC cell line during vegetative growth. The control and MLFkoSC cell lines were cultured in growth medium and then subjected to SDS-PAGE and Western blot analysis as described in Fig 1C. The blot was probed with anti-MLF, anti-CWP1, anti-MYB2, anti-WRKY, anti-PAX1, anti-CDK2, and anti-RAN antibodies, respectively. (G) Decrease of multiple gene expression by MLF knock down in the MLFkoSC cell line during vegetative growth. The control and MLFkoSC cell lines were cultured in growth medium and then subjected to quantitative real-time RT-PCR analysis using primers specific for *mlf*, *cwp1*, *cwp2*, *cwp3*, *myb2*, *wrky*, *pax1*, *cdk2*, *ran*, and 18S ribosomal RNA genes, respectively, as described in Fig 3F.
(PDF)

S7 Fig Decrease of *mlf*, *cwp1-3*, *myb2*, *wrky*, *pax1*, and *cdk2* expression by MLF knock down during encystation using strategy 2. (A) Cyst formation decreased by MLF knock down in the MLFkoSC cell line during encystation. The control and MLFkoSC cell lines were cultured in encystation medium for 24h (Enc) and then subjected to cyst count as described under “Methods” and Fig 1B. (B) Decrease of number of MVs by MLF knock down in the MLFkoSC cell line during encystation. The control and MLFkoSC cell lines were cultured in encystation medium and then subjected to immunofluorescence analysis using anti-MLF antibody for detection as described in Fig 3D. (C) Decrease of multiple gene expression by MLF knock down in the MLFkoSC cell line during encystation. The control and MLFko cell lines were cultured in encystation medium and then subjected to quantitative real-time RT-PCR analysis using primers specific for *mlf*, *cwp1*, *cwp2*, *cwp3*, *myb2*, *wrky*, *pax1*, *cdk2*, *ran*, and 18S ribosomal RNA genes, respectively, as described in Fig 3F.
(PDF)

S8 Fig Results from No-Cas9 control and No-HR-template control. (A) Diagrams of the pMLFko plasmid is the same as in Fig 2A. For No-Cas9 control, the pMLFko was transfected into trophozoites. SCR7 was added. After selection with puromycin, No-Cas9 control stable transfectants were established, although they grew slowly. The control cell line is trophozoites transfected with 5’Δ5N-Pac plasmid and selected with puromycin. (B) Genomic DNA was isolated from the No-Cas9 control cell line cultured in growth medium. PCR was performed using primers specific for *mlf* (PCR1), *pac* (PCR2) and *ran* genes, respectively, as described in Fig 3A. There was no product detected by PCR2. The PCR1 product was cloned into T vector, and sequenced. The sequence results obtained from the PCR1 product are the same as wild type nontransfected WB cells. (C) Real-time PCR was performed using genomic DNA and primers specific for *mlf*, *cwp1*, *cwp2*, and *ran* genes, respectively, as described in Fig 3B. (D) No change of the levels of MLF and CWP1 proteins in the No-Cas9 control. The control and No-Cas9 control cell lines were cultured in growth medium and then subjected to SDS-PAGE and Western blot analysis as described in Fig 1C. The blot was probed with anti-MLF, anti-CWP1, and anti-RAN antibodies, respectively. (E) Diagrams of the pgCas9, and pU6g plasmids are the same as in Fig 2A. For No-HR-template control, the pgCas9 and pU6g plasmids were transfected into trophozoites. SCR7 and puromycin were not added. The control cell line is wild type nontransfected WB trophozoites. (F) After transfection, genomic DNA was isolated from the No-HR-template control cell line cultured in growth medium. PCR was performed using primers specific for *mlf* (PCR1), *pac* (PCR2) and *ran* genes, respectively, as described in Fig 3A. There was no product detected by PCR2. The PCR1 product was cloned into T vector, and sequenced. The sequence results obtained from the PCR1 product are the same as wild type nontransfected WB cells. (G) Real-time PCR was performed using genomic DNA primers specific for *mlf*, *cwp1*, *cwp2*, and *ran* genes, respectively, as described in Fig 3B. (H) No change in the levels of MLF and CWP1 proteins in the No-HR-template control. The control and No-HR-template control cell lines were cultured in growth medium and then subjected to SDS-PAGE and Western blot analysis as described in Fig 1C. The blot was probed with anti-MLF, anti-CWP1, and anti-RAN antibodies, respectively.
(PDF)

S9 Fig Increase of MLF knock down efficiency during encystation using strategy 2 compared to strategy 1. (A) MLF knock down decreased cyst formation in the MLFkoSC cell line relative to the MLFko cell line during encystation. The MLFko and MLFkoSC cell lines were cultured in encystation medium for 24h (Enc) and then subjected to cyst count as described under “Materials and Methods” and Fig 1B. (B) Decrease of number of MVs by MLF knock-in the MLFkoSC cell line relative to the MLFko cell line during encystation. The MLFko and MLFkoSC cell lines were cultured in encystation medium and then subjected to immunofluorescence analysis using anti-MLF antibody for detection as described in Fig 3D. (C) Knock down of *mlf* gene decreased the levels of CWP1, MYB2, and other proteins in the MLFkoSC cell line relative to the MLFko cell line during encystation. The MLFko and MLFkoSC cell lines were cultured in encystation medium and then subjected to SDS-PAGE and Western blot analysis as described in Fig 1C. The blot was probed with anti-MLF, anti-CWP1, anti-MYB2, anti-WRKY, anti-PAX1, anti-CDK2, and anti-RAN antibodies, respectively. (D) Decrease of multiple gene expression by MLF knock down in the MLFkoSC cell line relative to the MLFko cell line during encystation. The MLFko and MLFkoSC cell lines were cultured in encystation medium and then subjected to quantitative real-time RT-PCR analysis using primers specific for *mlf*, *cwp1*, *cwp2*, *cwp3*, *myb2*, *wrky*, *pax1*, *cdk2*, *ran*, and 18S ribosomal RNA genes, respectively, as described in Fig 3F. (E) Cyst wall change by MLF knock down. The control, MLFko and MLFkoSC cell lines with puromycin selection were cultured in encystation medium and then subjected to immunofluorescence assay. The endogenous CWP1 protein was detected by anti-CWP1 antibody. The left panel shows that the CWP1 protein is localized to the cyst wall of the cyst. The middle panel shows the merge of DAPI and differential interference contrast images. The right panel shows the merged images. The diameter of cyst wall of the control, MLFko, and MLFkoSC cell line was estimated to be 415nm, 277nm, and 251nm, respectively.
(PDF)

S10 Fig Decrease of *cwp1*, *cwp2*, and *myb2* gene expression by MLF knock down during encystation using strategy 3. (A) Cyst formation decreased by MLF knock down in the Cas9MLFko cell line during encystation. The control and Cas9MLFko cell lines were cultured in encystation medium for 24h (Enc) and then subjected to cyst count as described under “Methods” and Fig 1B. (B) Knock down of *mlf* gene decreased the levels of MLF and CWP1 proteins in the Cas9MLFko cell line during encystation. The control and Cas9MLFko cell lines were cultured in encystation medium and then subjected to SDS-PAGE and Western blot analysis as described in Fig 1C. The blot was probed with anti-HA, anti-MLF, anti-CWP1, and anti-RAN antibodies, respectively. (C) Decrease of *mlf*, *cwp1*, and *cwp2* gene expression by MLF knock down in the Cas9MLFko cell line during encystation. The control and Cas9MLFko cell lines were cultured in encystation medium and then subjected to quantitative real-time RT-PCR analysis using primers specific for *mlf*, *cwp1*, *cwp2*, *ran*, and 18S ribosomal RNA genes, respectively, as described in Fig 3F. (D) Partial replacement of the *mlf* gene in two single clone populations of the Cas9MLFko cell line confirmed by PCR. Two single clone populations, clones 1 and 2, of the Cas9MLFko cell line were obtained using the single cell dilution method. Genomic DNA was isolated from Cas9MLFko clone 1, clone 2, and control cell lines cultured in growth medium. PCR was performed using primers specific for *mlf* (PCR1), *pac* (PCR2), and *ran* genes, respectively, as described in Fig 3A. (E) Partial disruption of *mlf* gene in two single clone populations of the Cas9MLFko cell line confirmed by real-time PCR. Real-time PCR was performed using genomic DNA and primers specific for *mlf*, *cwp1*, *cwp2*, and *ran* genes, respectively, as described in Fig 3B.
(PDF)

S11 Fig Results from two gRNA controls. (A) Diagrams of the pgCas9MLFko gRNA1 or 2 plasmid are the same as in Fig 2A. In construct pgCas9MLFko gRNA1, the gRNA 1, which targets *mlf* gene, has a different sequence compared with pgCas9MLFko in Fig 2A (also see S1 Table). In construct pgCas9MLFko gRNA2, the gRNA 2, which targets 20 mers of G, is a negative gRNA control (also see S1 Table). The pgCas9MLFko gRNA1 or 2 plasmid was transfected into trophozoites. An NHEJ inhibitor, SCR7, was added into the culture to increase HR. After selection with puromycin, Cas9MLFko gRNA1 or 2 stable transfectants were established, although the Cas9MLFko RNA2 transfectants grew slowly. (B) Partial replacement of the *mlf* gene with the *pac* gene in the Cas9MLFko gRNA1 cell line confirmed by PCR. Puromycin was kept in the Cas9MLFko gRNA1 and control cell lines as described in Fig 2A. Genomic DNA was isolated from Cas9MLFko gRNA1 and control cell lines cultured in growth medium (vegetative growth, Veg). PCR was performed using primers specific for *mlf* (PCR1), *pac* (PCR2), and *ran* genes, respectively, as described in Fig 3A. (C) Partial disruption of the *mlf* gene in the Cas9MLFko gRNA1 cell line confirmed by real-time PCR. Real-time PCR was performed using genomic DNA and primers specific for *mlf*, *cwp1*, *cwp2*, and *ran* genes, respectively, as described in Fig 3B. (D) Knock down of *mlf* gene decreased the levels of MLF and CWP1 proteins in the Cas9MLFko gRNA1 cell line during vegetative growth. The control and Cas9MLFko gRNA1 cell lines were cultured in growth medium and then subjected to SDS-PAGE and Western blot analysis as described in Fig 1C. The blot was probed with anti-MLF, anti-CWP1, and anti-RAN antibodies, respectively. (E) No replacement of the *mlf* gene with the *pac* gene in the Cas9MLFko gRNA2 cell line confirmed by PCR. Puromycin was kept in the Cas9MLFko gRNA2 and control cell lines as described in Fig 2A. Genomic DNA was isolated from Cas9MLFko gRNA2 and control cell lines cultured in growth medium (vegetative growth, Veg). PCR was performed using primers specific for *mlf* (PCR1), *pac* (PCR2), and *ran* genes, respectively, as described in Fig 3A. For Cas9MLFko gRNA2, there was no product detected by PCR2. The PCR1 product was cloned into T vector, and sequenced. The sequence results obtained from the PCR1 product are the same as wild type nontransfected WB cells. (F) No disruption of the *mlf* gene in the Cas9MLFko gRNA2 cell line confirmed by real-time PCR. Real-time PCR was performed using genomic DNA and primers specific for *mlf*, *cwp1*, *cwp2*, and *ran* genes, respectively, as described in Fig 3B. (G) No change of the levels of MLF and CWP1 proteins in the Cas9MLFko gRNA2 cell line during vegetative growth. The control and Cas9MLFko gRNA2 cell lines were cultured in growth medium and then subjected to SDS-PAGE and Western blot analysis as described in Fig 1C. The blot was probed with anti-MLF, anti-CWP1, and anti-RAN antibodies, respectively.
(PDF)

S12 Fig Decrease of *cwp1*, *cwp2*, and *myb2* gene expression by MLF knock down after the removal of puromycin during vegetative growth using strategy 3. (A) Partial disruption of the *mlf* gene in the Cas9MLFko–pu cell line confirmed by real-time PCR. Puromycin was removed from the Cas9MLFko cell line to obtain the Cas9MLFko–pu cell line as described in Fig 2C. Genomic DNA was isolated from Cas9MLFko–pu and control cell lines cultured in growth medium (vegetative growth, Veg). Real-time PCR was performed using genomic DNA and primers specific for *mlf*, *cwp1*, *cwp2*, and *ran* genes, respectively, as described in Fig 3B. (B) Cyst formation decreased by MLF knock down in the Cas9MLFko–pu cell line. The control and Cas9MLFko–pu cell lines were cultured in growth medium and then subjected to cyst count as described under “Materials and Methods” and Fig 1B. (C) Knock down of *mlf* gene decreased the levels of MLF and CWP1 proteins in the Cas9MLFko–pu cell line. The control and Cas9MLFko–pu cell lines were cultured in growth medium and then subjected to SDS-PAGE and Western blot analysis as described in Fig 1C. The blot was probed with anti-HA, anti-MLF, anti-CWP1, and anti-RAN antibodies, respectively. (D) Decrease of *mlf*, *cwp1*, and *cwp2* gene expression by MLF knock down in the Cas9MLFko–pu cell line. The control and Cas9MLFko–pu cell lines were cultured in growth medium and then subjected to quantitative real-time RT-PCR analysis using primers specific for *mlf*, *cwp1*, *cwp2*, *ran*, and 18S ribosomal RNA genes, respectively, as described in Fig 3F.
(PDF)

S13 Fig Decrease of *cwp1*, *cwp2*, and *myb2* gene expression by MLF knock down after the removal of puromycin during encystation using strategy 3. (A) Cyst formation decreased by MLF knock down in the Cas9MLFko–pu cell line during encystation. The control and Cas9MLFko–pu cell lines were cultured in encystation medium for 24h (Enc) and then subjected to cyst count as described under “Materials and Methods” and Fig 1B. (B) Knock down of *mlf* gene decreased the levels of MLF and CWP1 proteins in the Cas9MLFko–pu cell line during encystation. The control and Cas9MLFko–pu cell lines were cultured in encystation medium and then subjected to SDS-PAGE and Western blot analysis as described in Fig 1C. The blot was probed with anti-HA, anti-MLF, anti-CWP1, and anti-RAN antibodies, respectively. (C) Decrease of *mlf*, *cwp1*, and *cwp2* gene expression by MLF knock down in the Cas9MLFko–pu cell line during encystation. The control and Cas9MLFko–pu cell lines were cultured in encystation medium and then subjected to quantitative real-time RT-PCR analysis using primers specific for *mlf*, *cwp1*, *cwp2*, *ran*, and 18S ribosomal RNA genes, respectively, as described in Fig 3F.
(PDF)

S1 Table Oligonucleotides used for construction of plasmids and PCR. (PDF)
