# Supplementary material for: Development of CRISPR/Cas9-mediated gene disruption systems in Giardia lamblia
Source: PLoS One. 2019 Mar 11;14(3):e0213594. doi: 10.1371/journal.pone.0213594 (PMC6411161; doi:10.1371/journal.pone.0213594)

S1 Fig

A

```
Drosophila MLF    1  ----MSLFGALMGDFDDDLGLMNNHMNHTMNAMNMQMRSMNRLMNSFMPDPFMQVSPFDQ
Human MLF1        1  MFRMLNSSFEDDPFFSESILAHRENMRQMIRSFSEPFGR-DLLSISDGRGRAHNRRGHND
Human MLF2        1  MFRFMRDVEPEDPMFLMDPFAIHR--QHMSRMLSGGFGYSPFLSITDG--NMPGTRPASR
Giardia MLF       1  ----MSRTPNIEHANSSDGYNVRRPEEVTR--------------------PGERPQRR

Drosophila MLF   57  GFQQNALMERPQMPAMPAMGLFGMPMMPNFNRLLNADIGGNSGASFCQSTVMTMSSGPDG
Human MLF1       60  GEDSLTHTDVSSFQTMDQMVSNMRNYMQKLERNFGQLSVDPNGHSFCSSSVMTYSKIGDE
Human MLF2       57  RMQQAGAVSPFGMLGMSGGFMDMFGMMNDMIGNMEHMTAGGNCQTFSSSTVISYSNTGDG
Giardia MLF      35  YDDARDERDPPGFLAGFNPMASFQRMMDHMN-----DFFGSTRSIFGNMDKMFEDARHDP

Drosophila MLF  117  RPQIYQASTSTKTGPGGVRETRRTVQDSRTGVKKMAIGHHIGERAHIIEKEQDMRSGQLE
Human MLF1      120  PPKVFQASTQTRRAPGGIKETRKAMRDSDSGLEKMAIGHHIHDRAHVIKKSKNKKTGDEE
Human MLF2      117  APKVYQETSEMRSAPGGIRETRRTVRDSDSGLEQMSIGHHIRDRAHILQRSRNHRTGDQE
Giardia MLF      90  EGTHYVSSTSMCMLPGGVKEVKQRIVSNGKDVETHTR--AIGDKYISNKRERDIRTGRED

Drosophila MLF  177  ERQEFINLEEGEAEQFDREFTSRASRGAVQSRHHAGGMQAIMPARPAAHTSTLTIEPVED
Human MLF1      180  VNQEFINMNESDAHAFDEEWQSEVLKYKPGR-----------------------------
Human MLF2      177  ERQDYINLDESEAAAFDDEWRRETSRFRQQR-----------------------------
Giardia MLF     148  VSRDLYDVKEHEVDSFTRDFDDRMSKMPMRELFDR-------------------------

Drosophila MLF  237  DDDDDDDCVIQEQQPVRSSAGRHYSSAPTAPQNSIHPHPYAANPRRQQRAVKHFHTEDEA
Human MLF1      211  ---------HNLGNTRMRSVGHENPGSRELKRREKPQQSPAIEHGRRSNVLGDKLHIKGSS
Human MLF2      208  ---------P-LEFRRLESSGAG--GRRAEGPPRLAIQGPEDSPSRQSRRYDW--------
Giardia MLF     183  ------SHQNALFGGWDEYDRAYDTRRALQDRSSNRYDSRNDDGYGGRRRDDYHRDEDRY

Drosophila MLF  297  ASSYKAVKRGKKK---
Human MLF1      263  VKSNKK----------
Human MLF2           ----------------
Giardia MLF     237  GSDRYAGRHSSGNRRY
```

B

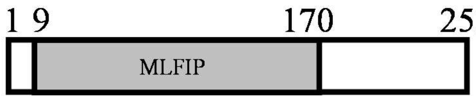

Supplement: S1 Fig — (A) Alignment of the MLF proteins. Specific sequence similarity search was performed against the GenBank database on NCBI's Web site using the BLASTP algorithm (http://blast.ncbi.nlm.nih.gov/Blast.cgi). This search identified similarity of Giardia MLF to Drosophila MLF human MLF1 and MLF2 in the GenBank database. Sequence of MLF proteins is analyzed by ClustalW 1.83, including Drosophila MLF human MLF1 and MLF2 (accession numbers are AFH08108.1, NP_071888.1, and NP_005430.1), and Giardia MLF (orf number 16424 in Giardia genome database). The gray boxes indicate the MLFIP domain of Giardia MLF predicted by pfam (http://pfam.sanger.ac.uk/). (B) Schematic representation of the Giardia MLF protein. The gray box indicates the MLFIP domain, as predicted by pfam. The residue numbers are shown. (PDF) [file pone.0213594.s001.pdf]
