# Supplementary figures and images for: Development of CRISPR/Cas9-mediated gene disruption systems in Giardia lamblia
Source: PLoS One. 2019 Mar 11;14(3):e0213594. doi: 10.1371/journal.pone.0213594 (PMC6411161; doi:10.1371/journal.pone.0213594)

S2 Fig

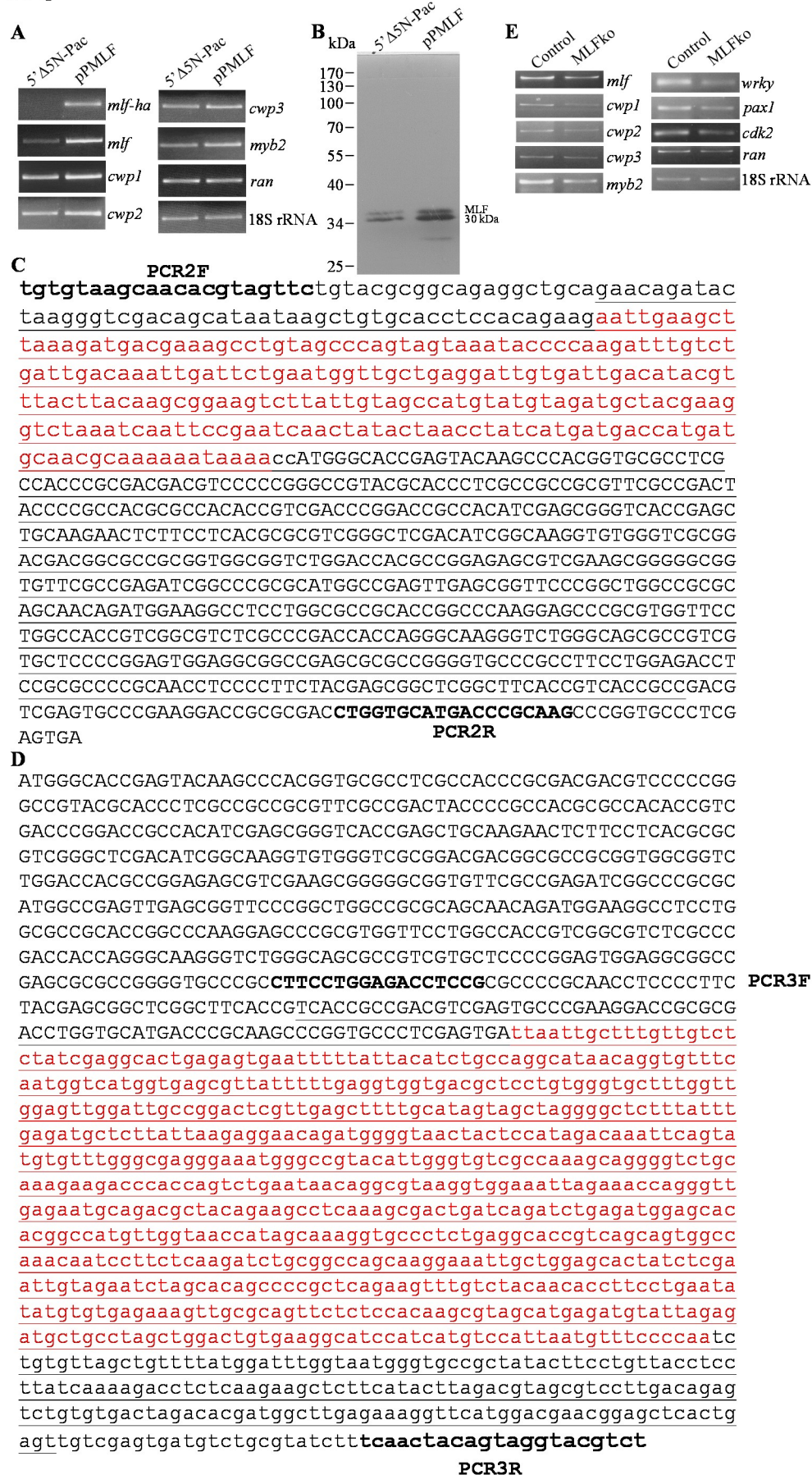

Supplement: S2 Fig — (A) RT-PCR analysis of gene expression in the MLF-overexpressing cell line. The 5’Δ5N-Pac and pPMLF stable transfectants were cultured in growth medium and then subjected to RT-PCR analysis. PCR was performed using primers specific for mlf-ha, mlf, cwp1, cwp2, cwp3, myb2, ran, and 18S ribosomal RNA genes, respectively. Similar levels of the ran mRNAs and 18S ribosomal RNA were detected. (B) Overexpression of MLF increased the levels of MLF proteins. The 5’Δ5N-Pac and pPMLF stable transfectants were cultured in growth medium and then subjected to SDS-PAGE and Western blot analysis. The blot was probed with anti-MLF antibody. The result is the same as in Fig 1C, but the whole gel is shown. (C) Replacement of the mlf gene with the pac gene in the MLFko cell line confirmed by PCR2 and sequencing. Genomic DNA was isolated from MLFko and control cell lines cultured in growth medium. PCR was performed using primers specific for pac (PCR2 in Fig 2A), which are PCR2F for bold region 1 and PCR2R for bold region 2, to verify the integration of pac gene into the correct region in genomic DNA. The sequence results obtained from the PCR2 product are shown as underlined letters. Capital letters indicate the coding sequence for pac gene, which starts at ATG and stops at TGA. This indicates the replacement of the mlf gene with the pac gene. The region used to clone the mlf 5’ region into the pMLFko plasmid for HR is shown in red, which is also between the sequence of MLF 5HF and MLF 5NR. The underlined and lower case letters, which are upstream and outside of the red region of MLF 5HF and MLF5NR, indicate that HR occurred in the sequence of mlf 5’ region and that the pac gene was integrated in the genomic DNA. Replacement of the mlf gene with the pac gene in the MLFkoSC and Cas9MLFko cell line was also confirmed by PCR2 and sequencing with the same sequencing results. (D) Replacement of the mlf gene with the pac gene in the MLFko cell line confirmed by PCR3 and sequencing. Genomic D [file pone.0213594.s002.pdf]

S3 Fig

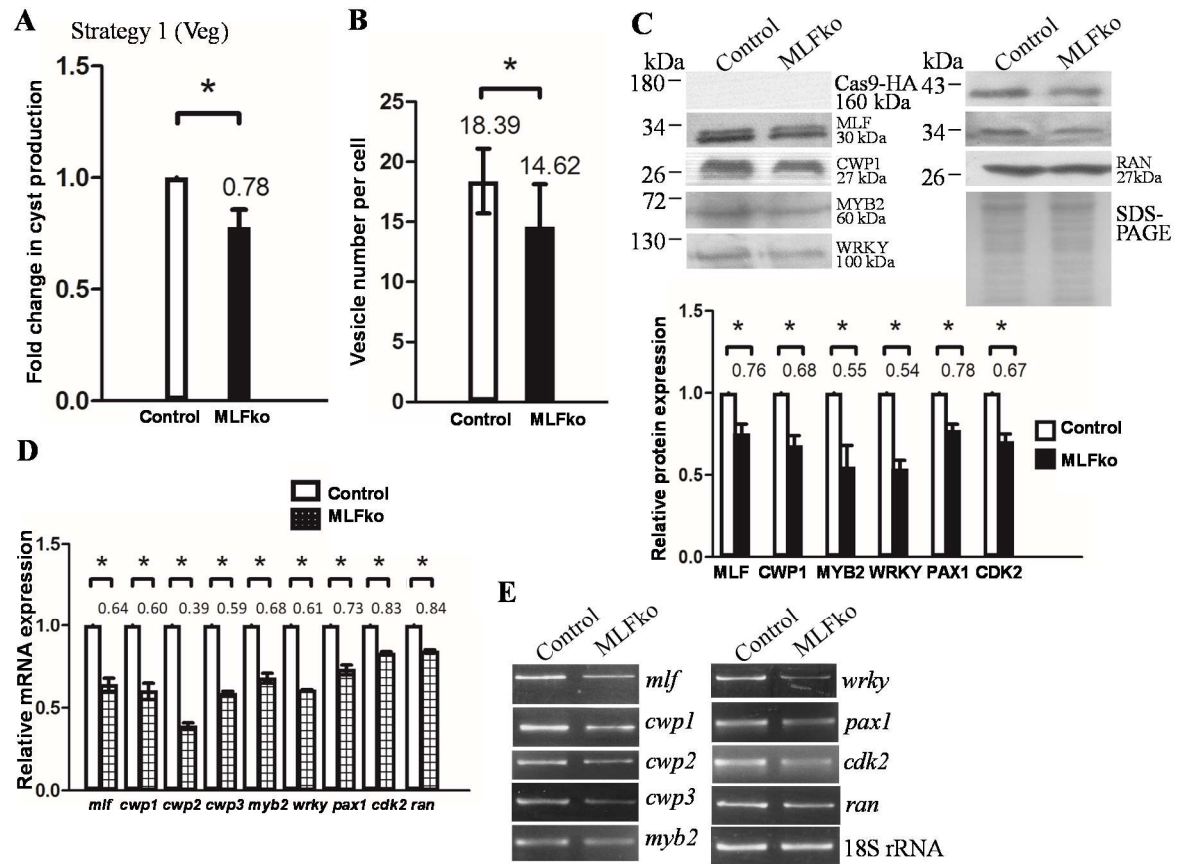

Supplement: S3 Fig — (A) Cyst formation decreased by MLF knock down in the MLFko cell line during vegetative growth. The control and MLFko cell lines were cultured in growth medium for 24h (Enc) and then subjected to cyst count as described under “Materials and Methods” and Fig 1B. (B) Decrease of number of MVs by MLF knock down in the MLFko cell line during vegetative growth. The control and MLFko cell lines were cultured in growth medium and then subjected to immunofluorescence analysis using anti-MLF antibody for detection as described in Fig 3D. (C) Knock down of mlf gene decreased the levels of CWP1, MYB2, and other proteins in the MLFko cell line during vegetative growth. The control and MLFko cell lines were cultured in growth medium and then subjected to SDS-PAGE and Western blot analysis, as described in Fig 1C. The blot was probed with anti-HA, anti-MLF, anti-CWP1, anti-MYB2, anti-WRKY, anti-PAX1, anti-CDK2, and anti-RAN antibodies, respectively. The intensity of bands from three Western blot assays was quantified using Image J. The ratio of each target protein over the loading control RAN is calculated. Fold change is calculated as the ratio of the difference between MLFko cell line and the control cell line, to which a value of 1 was assigned. Results are expressed as mean ± SD. *P<0.05. (D) Decrease of multiple gene expression by MLF knock down in the MLFko cell line during vegetative growth. The control and MLFko cell lines were cultured in growth medium and then subjected to quantitative real-time RT-PCR analysis using primers specific for mlf, cwp1, cwp2, cwp3, myb2, wrky, pax1, cdk2, ran, and 18S ribosomal RNA genes, respectively, as described in Fig 3F. (E) RT-PCR analysis of gene expression in the MLFko cell line during vegetative growth. The control and MLFko cell lines were cultured in growth medium and then subjected to RT-PCR analysis using primers specific for mlf, cwp1, cwp2, cwp3, myb2, wrky, pax1, cdk2, ran, and 18S ribosomal RNA genes, respectively. (PDF) [file pone.0213594.s003.pdf]

S4 Fig

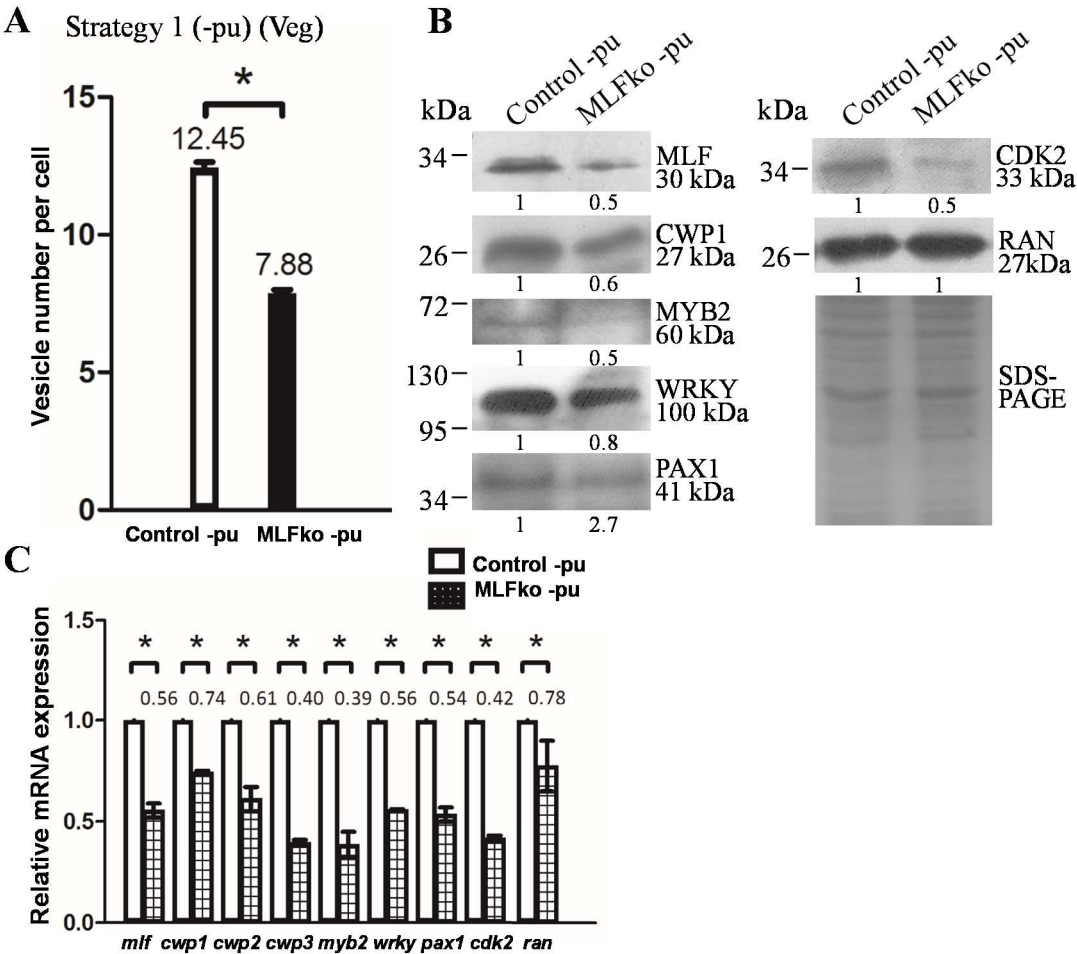

Supplement: S4 Fig — (A) Quantification of MVs in MLFko–pu and control–pu cell lines using Imaris software. *, P <0.05 (n = 200–300 cells/condition). (B) Knock down of mlf gene decreased the levels of CWP1, MYB2, and other proteins in the MLFko–pu cell line. The control–pu and MLFko–pu cell lines were cultured in growth medium and then subjected to SDS-PAGE and Western blot analysis, as described in Fig 1C. The blot was probed with anti-MLF, anti-CWP1, anti-MYB2, anti-WRKY, anti-PAX1, anti-CDK2, and anti-RAN antibodies, respectively. (C) Decrease of multiple gene expression by MLF knock down in the MLFko–pu cell line. The control–pu and MLFko–pu cell lines were cultured in growth medium and then subjected to quantitative real-time RT-PCR analysis using primers specific for mlf, cwp1, cwp2, cwp3, myb2, wrky, pax1, cdk2, ran, and 18S ribosomal RNA genes, respectively, as described in Fig 3F. (PDF) [file pone.0213594.s004.pdf]

S5 Fig

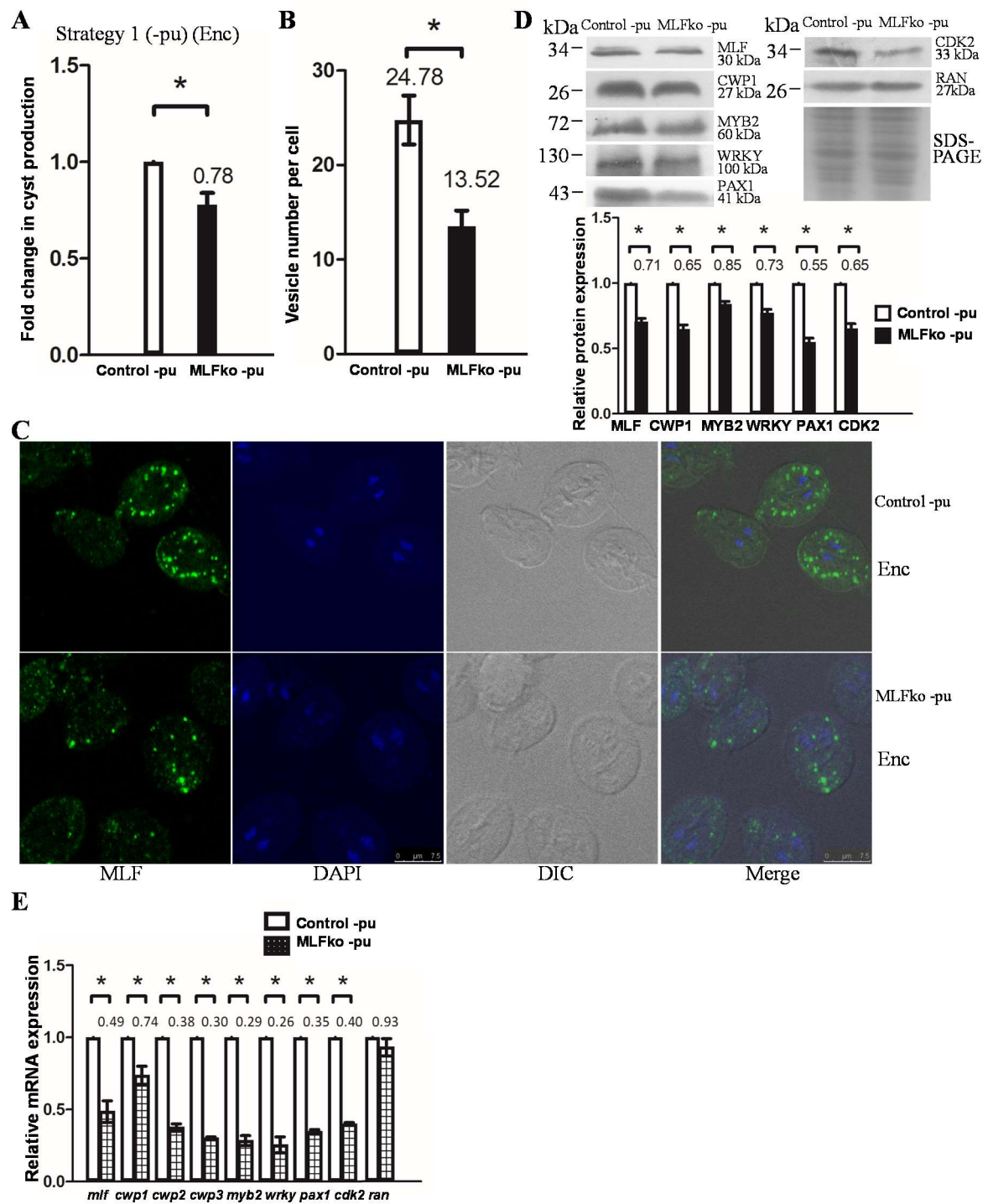

Supplement: S5 Fig — (A) Cyst formation decreased by MLF knock down in the MLFko–pu cell line during encystation. The control–pu and MLFko–pu cell lines were cultured in encystation medium for 24h (Enc) and then subjected to cyst count as described under “Methods” and Fig 1B. (B) Quantification of MVs in MLFko–pu and control–pu cell lines during encystation. The control–pu and MLFko–pu cell lines were cultured in encystation medium and then subjected to immunofluorescence analysis using anti-MLF antibody for detection as described in Fig 3D. (C) Decrease of number of MVs by MLF knock down in the MLFko–pu cell line during encystation. The control–pu and MLFko–pu cell lines were cultured in encystation medium and then subjected to immunofluorescence analysis using anti-MLF antibody for detection as described in Fig 4D. (D) Knock down of mlf gene decreased the levels of CWP1, MYB2, and other proteins in the MLFko–pu cell line during encystation. The control–pu and MLFko–pu cell lines were cultured in encystation medium and then subjected to SDS-PAGE and Western blot analysis, as described in Fig 1C. The blot was probed with anti-MLF, anti-CWP1, anti-MYB2, anti-WRKY, anti-PAX1, anti-CDK2, and anti-RAN antibodies, respectively. The intensity of bands from three Western blot assays was quantified using Image J. The ratio of each target protein over the loading control RAN is calculated. Fold change is calculated as the ratio of the difference between MLFko–pu cell line and the control–pu cell line, to which a value of 1 was assigned. Results are expressed as mean ± SD. *P<0.05. (E) Decrease of multiple gene expression by MLF knock down in the MLFko–pu cell line during encystation. The control–pu and MLFko–pu cell lines were cultured in encystation medium and then subjected to quantitative real-time RT-PCR analysis using primers specific for mlf, cwp1, cwp2, cwp3, myb2, wrky, pax1, cdk2, ran, and 18S ribosomal RNA genes, respectively, as described in Fig 3F. (PDF) [file pone.0213594.s005.pdf]

S6 Fig

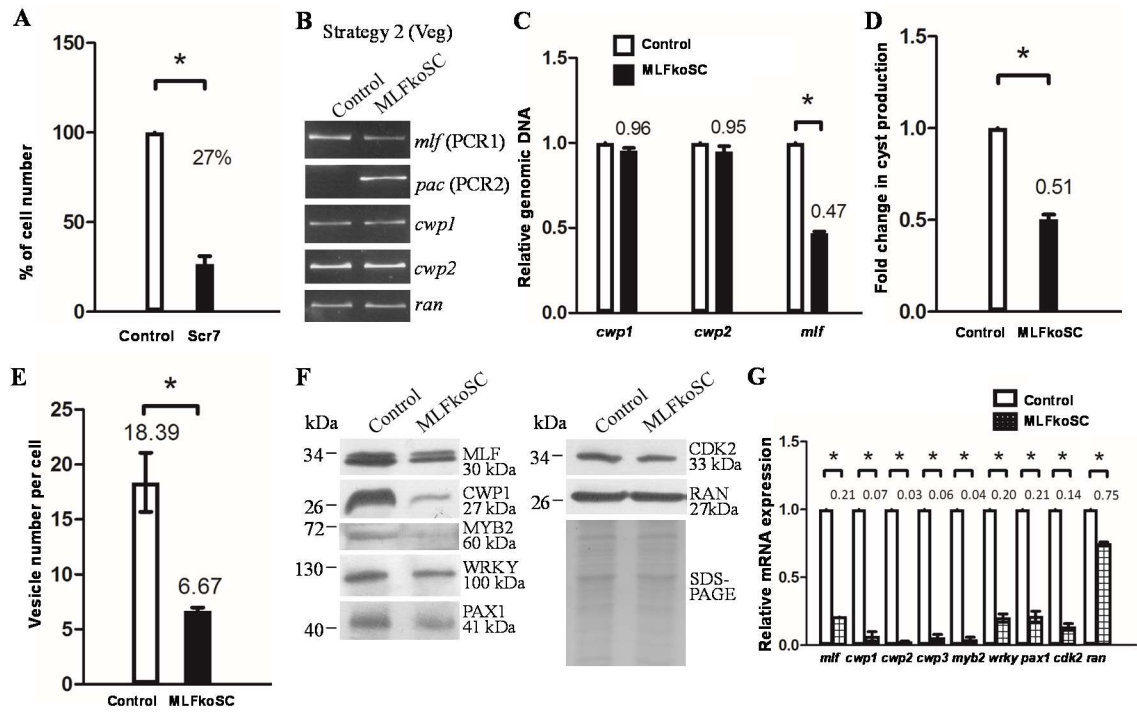

Supplement: S6 Fig — (A) Treatment with Scr7 decreased cell growth. The wild-type non-transfected WB cells were subcultured at an initial density of 1×10^6^ cells/ml in growth medium containing 33μM Scr7 for 24 h and then subjected to cell count. An equal volume of Me2SO was added to cultures as a negative control. The sum of total cells is expressed as a relative level over control. Values are shown as means ± S.E. of three independent experiments. *, P <0.05. (B) Partial replacement of the mlf gene with the pac gene in the MLFkoSC cell line confirmed by PCR. The pgCas9 and pMLFko constructs were transfected into trophozoites as described in Fig 2B. Puromycin was kept in the MLFkoSC and control cell lines. Genomic DNA was isolated from MLFkoSC and control cell lines cultured in growth medium (vegetative growth, Veg). PCR was performed using genomic DNA and primers specific for mlf (PCR1), pac (PCR3), cwp1, cwp2, and ran genes, respectively, as described in Fig 3A. (C) Partial disruption of the mlf gene in the MLFkoSC cell line confirmed by real-time PCR. Real-time PCR was performed using genomic DNA and primers specific for mlf, cwp1, cwp2, and ran genes, respectively, as described in Fig 3B. (D) MLF knock down decreased cyst formation in the MLFkoSC cell line during vegetative growth. The control and MLFkoSC cell lines were cultured in growth medium and then subjected to cyst count as described under “Methods” and Fig 1B. (E) Decrease of number of MVs by MLF knock down in the MLFkoSC cell line during vegetative growth. The control and MLFkoSC cell lines were cultured in growth medium and then subjected to immunofluorescence analysis using anti-MLF antibody for detection as described in Fig 3D. (F) Knock down of mlf gene decreased the levels of CWP1, MYB2, and other proteins in the MLFkoSC cell line during vegetative growth. The control and MLFkoSC cell lines were cultured in growth medium and then subjected to SDS-PAGE and Western blot analysis as described in Fig 1C. The blot was probe [file pone.0213594.s006.pdf]

S7 Fig

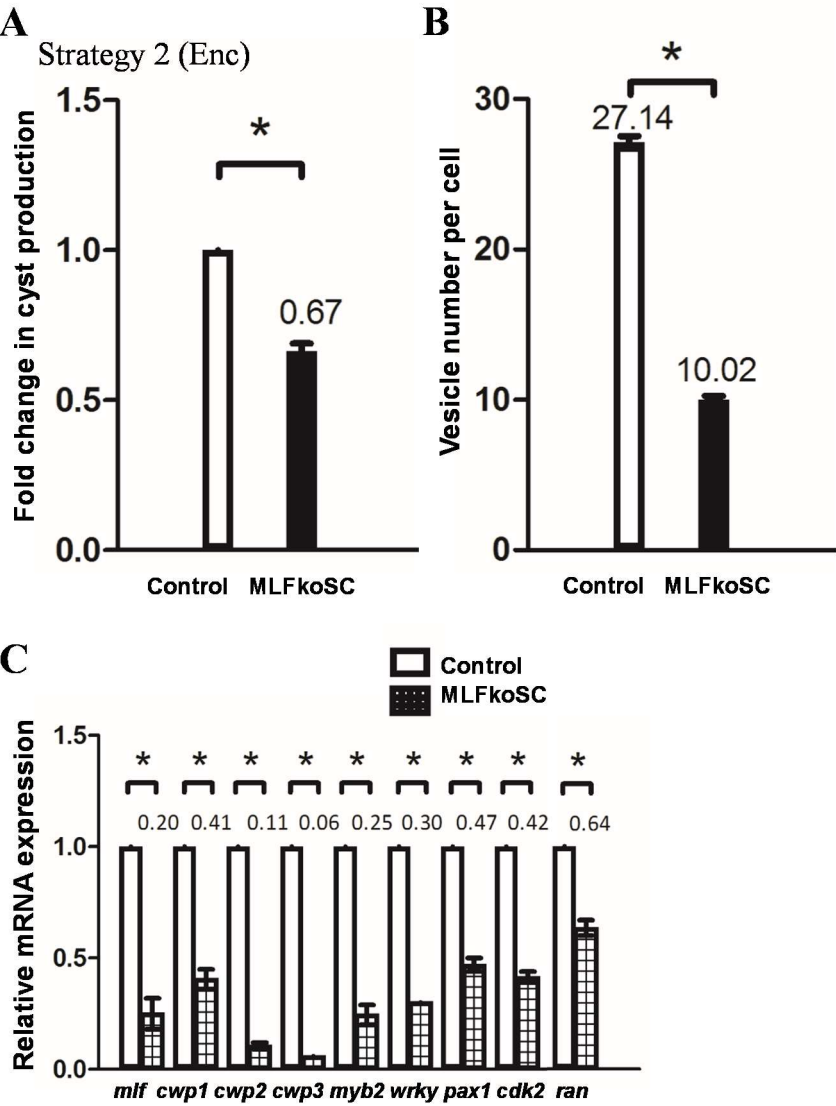

Supplement: S7 Fig — (A) Cyst formation decreased by MLF knock down in the MLFkoSC cell line during encystation. The control and MLFkoSC cell lines were cultured in encystation medium for 24h (Enc) and then subjected to cyst count as described under “Methods” and Fig 1B. (B) Decrease of number of MVs by MLF knock down in the MLFkoSC cell line during encystation. The control and MLFkoSC cell lines were cultured in encystation medium and then subjected to immunofluorescence analysis using anti-MLF antibody for detection as described in Fig 3D. (C) Decrease of multiple gene expression by MLF knock down in the MLFkoSC cell line during encystation. The control and MLFko cell lines were cultured in encystation medium and then subjected to quantitative real-time RT-PCR analysis using primers specific for mlf, cwp1, cwp2, cwp3, myb2, wrky, pax1, cdk2, ran, and 18S ribosomal RNA genes, respectively, as described in Fig 3F. (PDF) [file pone.0213594.s007.pdf]

S8 Fig

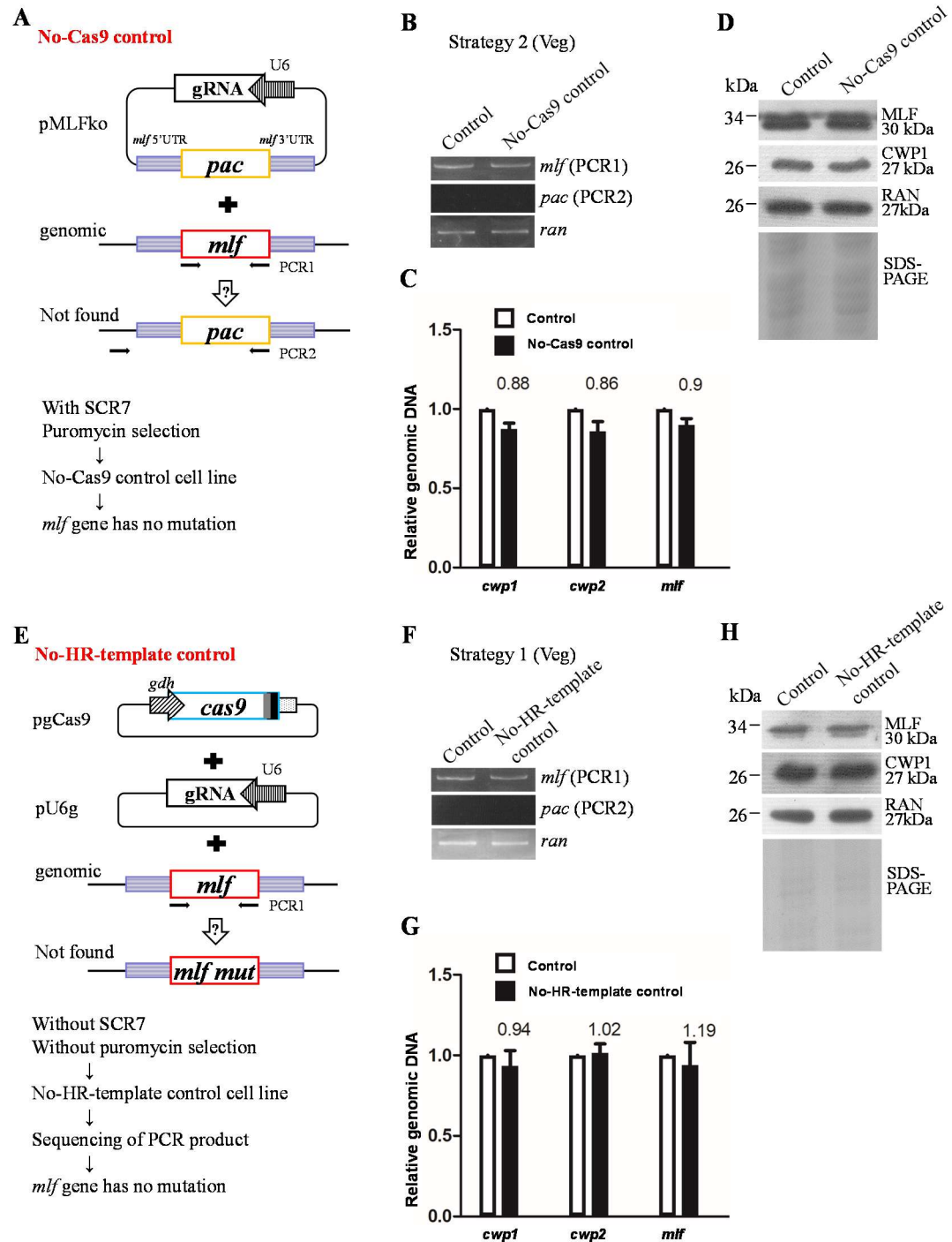

Supplement: S8 Fig — (A) Diagrams of the pMLFko plasmid is the same as in Fig 2A. For No-Cas9 control, the pMLFko was transfected into trophozoites. SCR7 was added. After selection with puromycin, No-Cas9 control stable transfectants were established, although they grew slowly. The control cell line is trophozoites transfected with 5’Δ5N-Pac plasmid and selected with puromycin. (B) Genomic DNA was isolated from the No-Cas9 control cell line cultured in growth medium. PCR was performed using primers specific for mlf (PCR1), pac (PCR2) and ran genes, respectively, as described in Fig 3A. There was no product detected by PCR2. The PCR1 product was cloned into T vector, and sequenced. The sequence results obtained from the PCR1 product are the same as wild type nontransfected WB cells. (C) Real-time PCR was performed using genomic DNA and primers specific for mlf, cwp1, cwp2, and ran genes, respectively, as described in Fig 3B. (D) No change of the levels of MLF and CWP1 proteins in the No-Cas9 control. The control and No-Cas9 control cell lines were cultured in growth medium and then subjected to SDS-PAGE and Western blot analysis as described in Fig 1C. The blot was probed with anti-MLF, anti-CWP1, and anti-RAN antibodies, respectively. (E) Diagrams of the pgCas9, and pU6g plasmids are the same as in Fig 2A. For No-HR-template control, the pgCas9 and pU6g plasmids were transfected into trophozoites. SCR7 and puromycin were not added. The control cell line is wild type nontransfected WB trophozoites. (F) After transfection, genomic DNA was isolated from the No-HR-template control cell line cultured in growth medium. PCR was performed using primers specific for mlf (PCR1), pac (PCR2) and ran genes, respectively, as described in Fig 3A. There was no product detected by PCR2. The PCR1 product was cloned into T vector, and sequenced. The sequence results obtained from the PCR1 product are the same as wild type nontransfected WB cells. (G) Real-time PCR was performed using genomic DNA primers s [file pone.0213594.s008.pdf]

S9 Fig

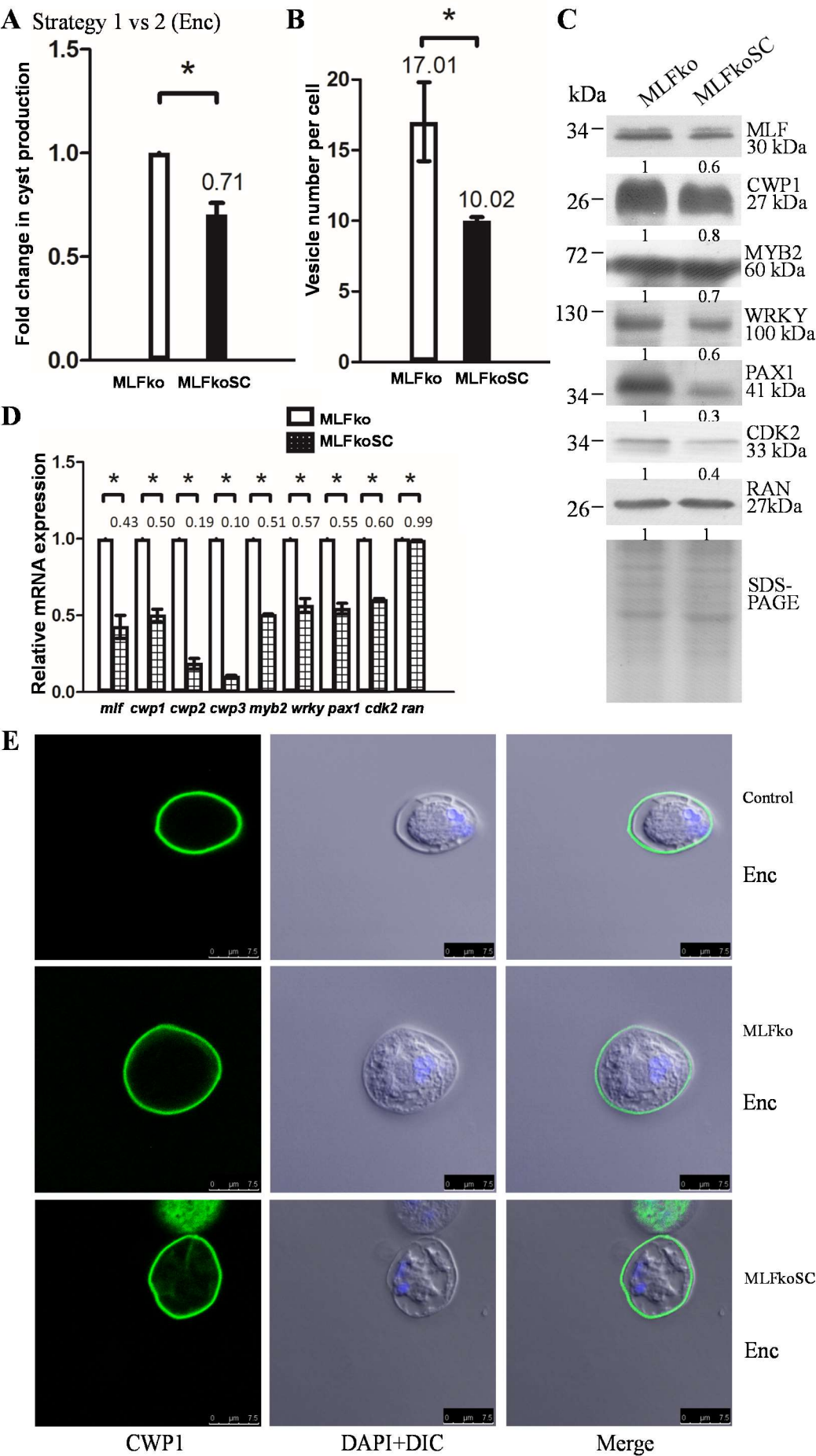

Supplement: S9 Fig — (A) MLF knock down decreased cyst formation in the MLFkoSC cell line relative to the MLFko cell line during encystation. The MLFko and MLFkoSC cell lines were cultured in encystation medium for 24h (Enc) and then subjected to cyst count as described under “Materials and Methods” and Fig 1B. (B) Decrease of number of MVs by MLF knock-in the MLFkoSC cell line relative to the MLFko cell line during encystation. The MLFko and MLFkoSC cell lines were cultured in encystation medium and then subjected to immunofluorescence analysis using anti-MLF antibody for detection as described in Fig 3D. (C) Knock down of mlf gene decreased the levels of CWP1, MYB2, and other proteins in the MLFkoSC cell line relative to the MLFko cell line during encystation. The MLFko and MLFkoSC cell lines were cultured in encystation medium and then subjected to SDS-PAGE and Western blot analysis as described in Fig 1C. The blot was probed with anti-MLF, anti-CWP1, anti-MYB2, anti-WRKY, anti-PAX1, anti-CDK2, and anti-RAN antibodies, respectively. (D) Decrease of multiple gene expression by MLF knock down in the MLFkoSC cell line relative to the MLFko cell line during encystation. The MLFko and MLFkoSC cell lines were cultured in encystation medium and then subjected to quantitative real-time RT-PCR analysis using primers specific for mlf, cwp1, cwp2, cwp3, myb2, wrky, pax1, cdk2, ran, and 18S ribosomal RNA genes, respectively, as described in Fig 3F. (E) Cyst wall change by MLF knock down. The control, MLFko and MLFkoSC cell lines with puromycin selection were cultured in encystation medium and then subjected to immunofluorescence assay. The endogenous CWP1 protein was detected by anti-CWP1 antibody. The left panel shows that the CWP1 protein is localized to the cyst wall of the cyst. The middle panel shows the merge of DAPI and differential interference contrast images. The right panel shows the merged images. The diameter of cyst wall of the control, MLFko, and MLFkoSC cell line was estimated to [file pone.0213594.s009.pdf]

S10 Fig

**A** Strategy 3 (Enc)

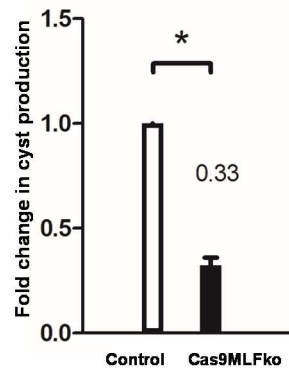

**B**

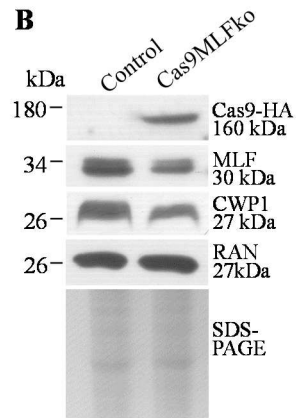

**D**

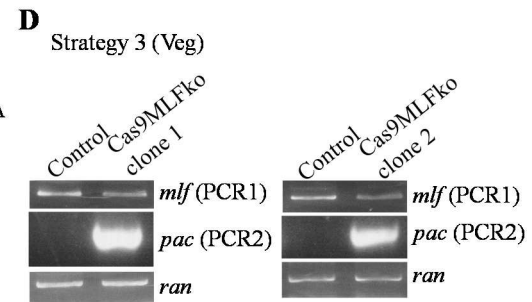

**C**

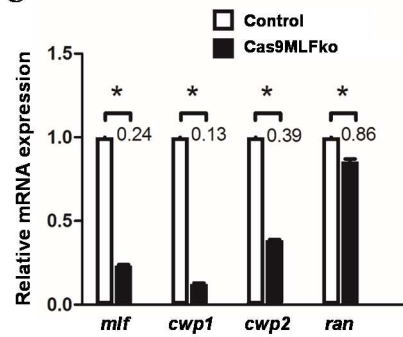

**E**

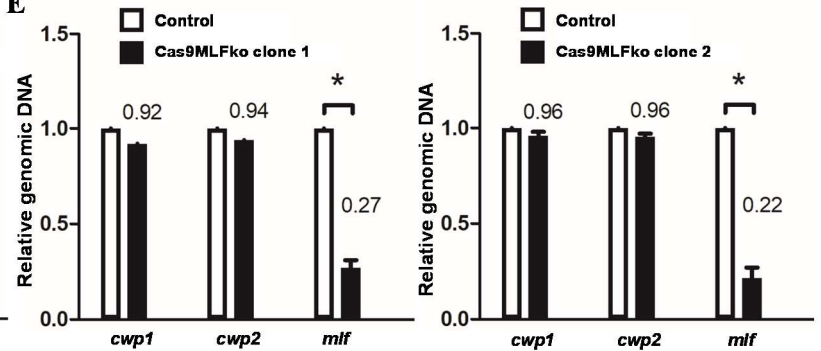

Supplement: S10 Fig — (A) Cyst formation decreased by MLF knock down in the Cas9MLFko cell line during encystation. The control and Cas9MLFko cell lines were cultured in encystation medium for 24h (Enc) and then subjected to cyst count as described under “Methods” and Fig 1B. (B) Knock down of mlf gene decreased the levels of MLF and CWP1 proteins in the Cas9MLFko cell line during encystation. The control and Cas9MLFko cell lines were cultured in encystation medium and then subjected to SDS-PAGE and Western blot analysis as described in Fig 1C. The blot was probed with anti-HA, anti-MLF, anti-CWP1, and anti-RAN antibodies, respectively. (C) Decrease of mlf, cwp1, and cwp2 gene expression by MLF knock down in the Cas9MLFko cell line during encystation. The control and Cas9MLFko cell lines were cultured in encystation medium and then subjected to quantitative real-time RT-PCR analysis using primers specific for mlf, cwp1, cwp2, ran, and 18S ribosomal RNA genes, respectively, as described in Fig 3F. (D) Partial replacement of the mlf gene in two single clone populations of the Cas9MLFko cell line confirmed by PCR. Two single clone populations, clones 1 and 2, of the Cas9MLFko cell line were obtained using the single cell dilution method. Genomic DNA was isolated from Cas9MLFko clone 1, clone 2, and control cell lines cultured in growth medium. PCR was performed using primers specific for mlf (PCR1), pac (PCR2), and ran genes, respectively, as described in Fig 3A. (E) Partial disruption of mlf gene in two single clone populations of the Cas9MLFko cell line confirmed by real-time PCR. Real-time PCR was performed using genomic DNA and primers specific for mlf, cwp1, cwp2, and ran genes, respectively, as described in Fig 3B. (PDF) [file pone.0213594.s010.pdf]

**S11 Fig**

**A** Two gRNA controls

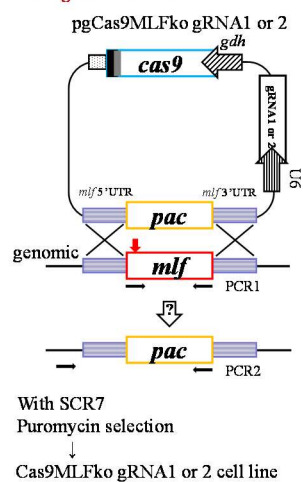

**B** Strategy 3 (Veg)

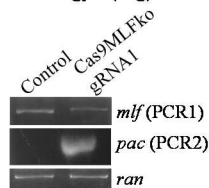

**C**

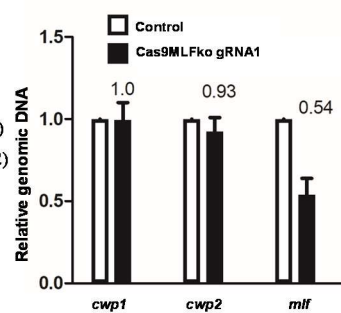

## D

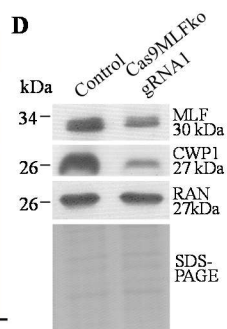

**E** Strategy 3 (Veg)

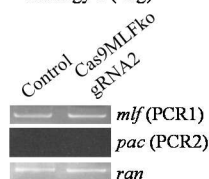**F**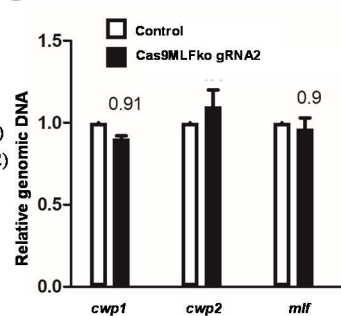

**G**

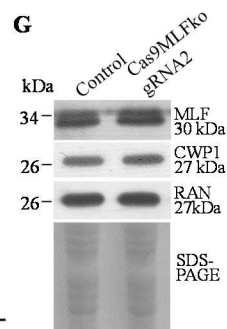

Supplement: S11 Fig — (A) Diagrams of the pgCas9MLFko gRNA1 or 2 plasmid are the same as in Fig 2A. In construct pgCas9MLFko gRNA1, the gRNA 1, which targets mlf gene, has a different sequence compared with pgCas9MLFko in Fig 2A (also see S1 Table). In construct pgCas9MLFko gRNA2, the gRNA 2, which targets 20 mers of G, is a negative gRNA control (also see S1 Table). The pgCas9MLFko gRNA1 or 2 plasmid was transfected into trophozoites. An NHEJ inhibitor, SCR7, was added into the culture to increase HR. After selection with puromycin, Cas9MLFko gRNA1 or 2 stable transfectants were established, although the Cas9MLFko RNA2 transfectants grew slowly. (B) Partial replacement of the mlf gene with the pac gene in the Cas9MLFko gRNA1 cell line confirmed by PCR. Puromycin was kept in the Cas9MLFko gRNA1 and control cell lines as described in Fig 2A. Genomic DNA was isolated from Cas9MLFko gRNA1 and control cell lines cultured in growth medium (vegetative growth, Veg). PCR was performed using primers specific for mlf (PCR1), pac (PCR2), and ran genes, respectively, as described in Fig 3A. (C) Partial disruption of the mlf gene in the Cas9MLFko gRNA1 cell line confirmed by real-time PCR. Real-time PCR was performed using genomic DNA and primers specific for mlf, cwp1, cwp2, and ran genes, respectively, as described in Fig 3B. (D) Knock down of mlf gene decreased the levels of MLF and CWP1 proteins in the Cas9MLFko gRNA1 cell line during vegetative growth. The control and Cas9MLFko gRNA1 cell lines were cultured in growth medium and then subjected to SDS-PAGE and Western blot analysis as described in Fig 1C. The blot was probed with anti-MLF, anti-CWP1, and anti-RAN antibodies, respectively. (E) No replacement of the mlf gene with the pac gene in the Cas9MLFko gRNA2 cell line confirmed by PCR. Puromycin was kept in the Cas9MLFko gRNA2 and control cell lines as described in Fig 2A. Genomic DNA was isolated from Cas9MLFko gRNA2 and control cell lines cultured in growth medium (vegetative growth, Veg). [file pone.0213594.s011.pdf]

S12 Fig

**A** Strategy 3 (Veg)

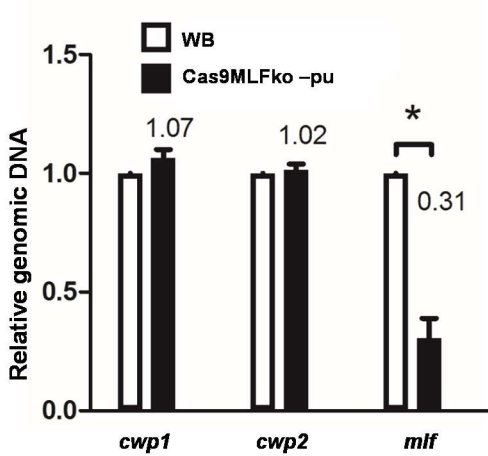

**B**

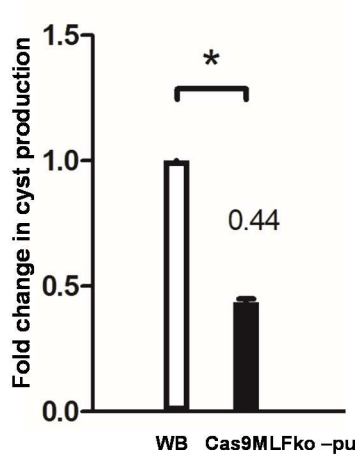

**C**

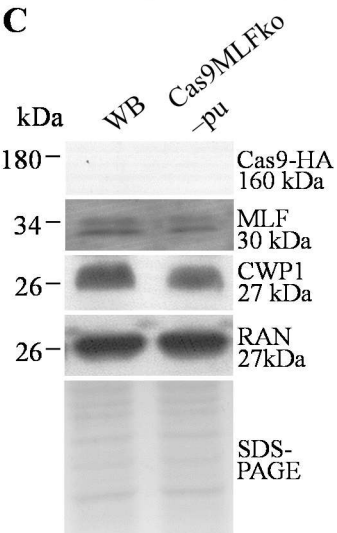

**D**

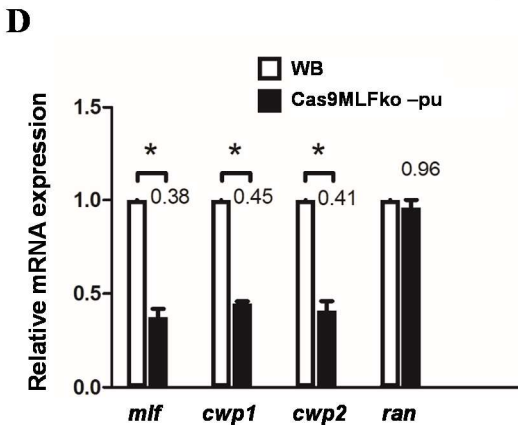

Supplement: S12 Fig — (A) Partial disruption of the mlf gene in the Cas9MLFko–pu cell line confirmed by real-time PCR. Puromycin was removed from the Cas9MLFko cell line to obtain the Cas9MLFko–pu cell line as described in Fig 2C. Genomic DNA was isolated from Cas9MLFko–pu and control cell lines cultured in growth medium (vegetative growth, Veg). Real-time PCR was performed using genomic DNA and primers specific for mlf, cwp1, cwp2, and ran genes, respectively, as described in Fig 3B. (B) Cyst formation decreased by MLF knock down in the Cas9MLFko–pu cell line. The control and Cas9MLFko–pu cell lines were cultured in growth medium and then subjected to cyst count as described under “Materials and Methods” and Fig 1B. (C) Knock down of mlf gene decreased the levels of MLF and CWP1 proteins in the Cas9MLFko–pu cell line. The control and Cas9MLFko–pu cell lines were cultured in growth medium and then subjected to SDS-PAGE and Western blot analysis as described in Fig 1C. The blot was probed with anti-HA, anti-MLF, anti-CWP1, and anti-RAN antibodies, respectively. (D) Decrease of mlf, cwp1, and cwp2 gene expression by MLF knock down in the Cas9MLFko–pu cell line. The control and Cas9MLFko–pu cell lines were cultured in growth medium and then subjected to quantitative real-time RT-PCR analysis using primers specific for mlf, cwp1, cwp2, ran, and 18S ribosomal RNA genes, respectively, as described in Fig 3F. (PDF) [file pone.0213594.s012.pdf]

**S13 Fig**

**A** Strategy 3 (Enc)

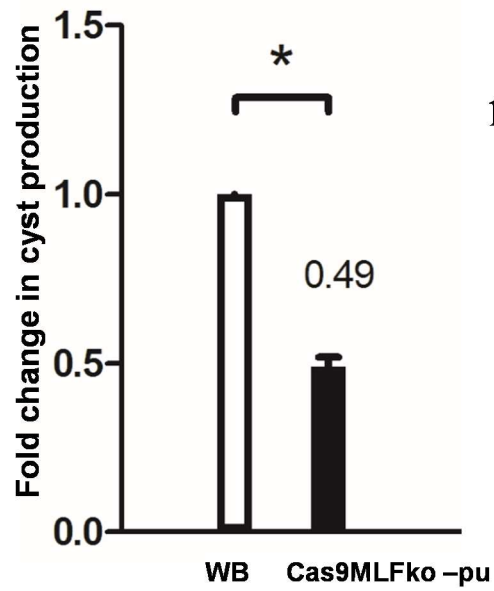

**B**

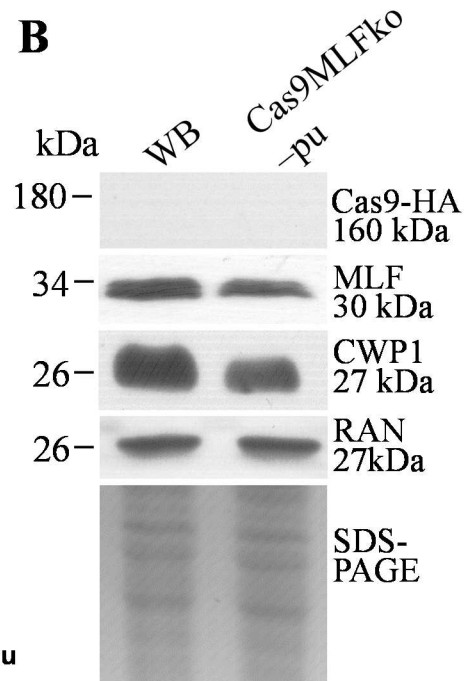

**C**

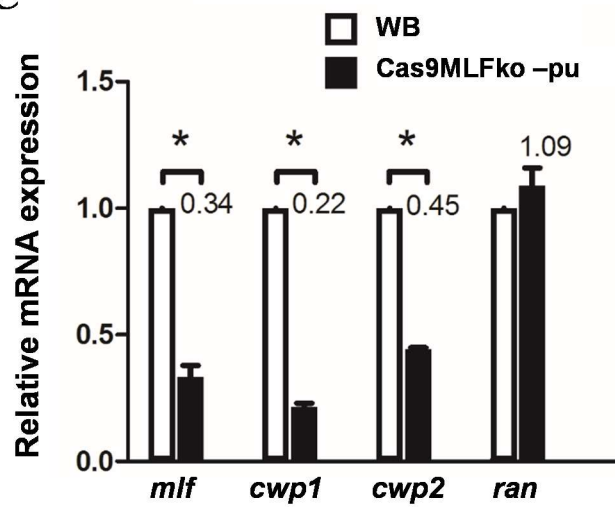

Supplement: S13 Fig — (A) Cyst formation decreased by MLF knock down in the Cas9MLFko–pu cell line during encystation. The control and Cas9MLFko–pu cell lines were cultured in encystation medium for 24h (Enc) and then subjected to cyst count as described under “Materials and Methods” and Fig 1B. (B) Knock down of mlf gene decreased the levels of MLF and CWP1 proteins in the Cas9MLFko–pu cell line during encystation. The control and Cas9MLFko–pu cell lines were cultured in encystation medium and then subjected to SDS-PAGE and Western blot analysis as described in Fig 1C. The blot was probed with anti-HA, anti-MLF, anti-CWP1, and anti-RAN antibodies, respectively. (C) Decrease of mlf, cwp1, and cwp2 gene expression by MLF knock down in the Cas9MLFko–pu cell line during encystation. The control and Cas9MLFko–pu cell lines were cultured in encystation medium and then subjected to quantitative real-time RT-PCR analysis using primers specific for mlf, cwp1, cwp2, ran, and 18S ribosomal RNA genes, respectively, as described in Fig 3F. (PDF) [file pone.0213594.s013.pdf]
