# Supplementary material for: Development of CRISPR/Cas9-mediated gene disruption systems in Giardia lamblia
Source: PLoS One. 2019 Mar 11;14(3):e0213594. doi: 10.1371/journal.pone.0213594 (PMC6411161; doi:10.1371/journal.pone.0213594)
Supplement: S1 Table — (PDF) [file pone.0213594.s014.pdf]

S1 Table. Oligonucleotides used for construction of plasmids and PCR.

Name	Sequence (5'--->3')
mlfF (PCR1F)	CACCATGAGTAGAACGCCAAAC
mlfR (PCR1R)	GTAGCGACGATTACCGGA
mlfHAF	AGCTTTTCGACCGAAGTCACC
HAR	AGCGTAATCTGGAACATCGTATGGGTA
cwp1F	ATGATGCTCGCTCTCCTT
cwp1R	TCAAGGCGGGGTGAGGCA
cwp2F	ATGATCGCAGCCCTTGTTCTA
cwp2R	CCTTCTGCGGACAATAGGCTT
cwp3F	ATGTTTTCTCTGCTTCTTCT
cwp3R	TCTGTAGTAGGGCGGCTGTA
myb2F	ATGTTACCGGTACCTTCTCAGC
myb2R	GGGTAGCTTCTCACGGGGAAG
wrkyF	ATGAAAGAAGGATCCCTGCA
wrkyR	AGAGTAAACGTTAACCATCGG
pax1F	CACCATGTCCGAGTATGATGAGCA
pax1R	ATACACATCAACGTCCATCT
cdk2F	CACCATGACTGACCCCTTGAAC
cdk2R	CTTTGCAAAGTACGGATGCTTG
MLFgF	CGTGATGAGAGAGATCCGCCGTTTTAGAGCTAGAAATAGCAAG
MLFgR	GGCGGACGCGTAATTCAAAAAAGCACGGACTC
ranF	ATGTCTGACCCAATCAGC
ranR	TCAATCATCGTCGGGAAG
mlfrealF	AGCATTTTTGGAAACATGGAT
mlfrealR	AAGCAGCGCATAGTCTCAAAC
cwp1realF	AACGCTCTCACAGGCTCCAT
cwp1realR	AGGTGGAGCTCCTTGAGAAATTG
cwp2realF	TAGGCTGCTTCCCACTTTTGAG
cwp2realR	CGGGCCCGCAAGGT
cwp3realF	GCAAATTGGATGCCAAACAA

cwp3realR	GACTCCGATCCAGTCGCAGTA
myb2realF	TCCCTAATGACGCCAAACG
myb2realR	AGCACGCAGAGGCCAAGT
wrkyrealF	ATTGACGCAGGGCATTCG
wrkyrealR	CGAAGGGATTTTGCTGGTAGAC
pax1realF	GGCTCCCGATAATACTATCACTCTTG
pax1realR	GGATCAGAAGCCGGTCCAT
cdk2realF	GCGTCTTGCACCGTGATCTA
cdk2realR	TTAAGGAGCCCAGAGCTGGTAA
ranrealF	TCGTCCTCGTCGGAAACAA
ranrealR	AACTGTCTGGGTGCGGATCT
18SrealF	AAGACCGCCTCTGTCAATCAA
18SrealR	GTTTACGGCCGGGAATACG
MLF 5HF	AATTGAAGCTTAAAGATGACG
MLF 5NR	GGCGGCCATGGTTTTATTTTTTGCGTTGCATC
MLF 3XF	GGCGGCTCGAGTGACTAATTGCTTTGTTGTCTCTA
MLF 3KR	GGCGGGGTACCTTGGGGAAACATTAATGGACA
MLF-guide	GAGAGCGGGTACCCTAGCTTATTGAAAAAGCGAGAGGCCATTGAGATGACTCGCCTGATTGCAATAGCAAACAGTGTCTATAGTCTAATTGTGGACAACAGAGGGCTTATTGCAACGTTGATGACCAAGTTCAACAAGGGCGTCGCCTCCTATGAGCAGGTCATTCGGAATTTCGACGACCGGTAGCGTCCCCAGAGTAAACCATTTTAAATTGAAATAGGCGGTTGGAAATAAAAGCGCGCC**CGTGATGAGAGAGATCC*****GCC***GTTTTAGAGCTAGAAATAGCAAGTTAAAATAAGGCTAGTCCGTTATCAACTTGAAAAAGTGGCACCGAGTCGGTGCTTTTTTGAATTCGAGAGCG, underlined region is U6 promoter sequence (Genebank accession number JX416864 is the sequence of the U6 small nuclear RNA, the U6 promoter is 230 bp upstream of the sequence of U6 small nuclear RNA), bold region is for annealing, underlined and bold region is upstream 3nt of PAM, the other region is scaffold RNA, the gRNA targets to nt 115-134 of *mlf* gene.
Cas9NF	GGGCGCCATGGATGGACAAGAAGTACTCCATT
Cas9XR	GGCGCTCGAGAAAGTCGATGGGGTTTTTTTC
neomF	TCTCGTCGTGACCCAcGGCGATGCCTGCTTG, the mutated sequence in the *neo* gene is shown in lower case
neoXR	GGCCGCTCGAGTCAGAAGAACTCGTCAAGAAG

neomR	CAAGCAGGCATCGCCgTGGGTCACGACGAGA, the mutated sequence in the *neo* gene is shown in lower case
neoNF	GGCCGCCATGGATGATTGAACAAGATGGATTG
MLFNF	GGCGGCTAGCTGTGTAAGCAACACGTAGTTC
MLFMR	GGCGACGCGTGTAGCGACGATTACCGGA
Cas9insF	ATCACGCGTGATCCCTAGGGATCATCGATGACCACAAATAACGCCTTTAATTAC
Cas9insR	CGCTAGCGATCGCGGCCGCGATCTCTAGAAAGCTTGAGTATTCTATAGTGTCAC
mlf53UNF	GGCGGGCGGCCGCAGCTTAAAGATGACGAAAGCC
mlf53UMR	GGCGGACGCGTAATTCAAAAAAGCACCGACTC
PCR2F	TGTGTAAGCAACACGTAGTTC
PCR2R	CTTGCGGGTCATGCACCAG
PCR3F	CTTCCTGGAGACCTCCG
PCR3R	AGACGTACCTACTGTAGTTGA
Cas9CXK	GGCGGCCTCGAGGCGAAAGGATATAAAGAGGTCAAAAAAGACCTCATCATTAAGCTTCCCAAGTACTCTCTCTTTGAGCTTGAAAACGGCCGGAAACGAATGCTCGCTAGTGCGGGCGAGCTGCAGAAAGGTAACGAGCTGGCACTGCCCTCTAAATACGTTAATTTCTTGTATCTGGCCAGCCACTATGAAAAGCTCAAAGGGTCTCCCGAAGATAATGAGCAGAAGCAGCTGTTCGTGGAACAACACAAACACTACCTTGATGAGATCATCGAGCAAATAAGCGAATTCTCCAAAAGAGTGATCCTCGCCGACGCTAACCTCGATAAGGTGCTTTCTGCTTACAATAAGCACAGGGATAAGCCCATCAGGGAGCAGGCAGAAAACATTATCCACTTGTTTACTCTGACCAACTTGGGCGCGCCTGCAGCCTTCAAGTACTTCGACACCACCATAGACAGAAAGCGGTACACCTCTACAAAGGAGGTCCTGGACGCCACACTGATTCATCAGTCAATTACGGGGCTCTATGAAACAAGAATCGACCTCTCTCAGCTCGGTGGAGAC**AAGAAGAAGCGCAAGGTG**TACCCATACGATGTTCCAGATTACGCT**TGATCTAGAGCAGGCCTTTGATGACTGCTTTTTCTGCCGCGTTGTTTCGACCGTCCTGGACTTCCACCTGCAGGTAGTAAATGCGCTATCTTTCGGGCTAAGAAAATAATCGGTGGATGGCGGCGGGACCGACTCTTATCTCTCCACTGCGCTACACCGGCATAACTCCACTTGCAACTGACTTTCTGGGGATAAGCTATAAGGCGCAGCGTGATGAAGACGGCGCTCTTCTAGCGGTTCGTTAC**ATCGATGGTACCGGCGGC, gray region is Cas9 coding region near stop codon, gray and bold region is nuclear localization signal coding region, underlined region is HA coding region, underlined and bold region is 3' untranslated region of *ran* gene
MLFgFnew	CCAGAAGAGGTTACAAGGCCGTTTTAGAGCTAGAAATAGC, the gRNA targets to nt 61-80 of mlf gene.

control gF	CCCCCCCCCCCCCCCCCCCCGTTTTAGAGCTAGAAATAGC, the gRNA targets to 20 of G.
U6R	GGCGCGCTTTTATTTCCAACC
